# Linalool acts as a fast and reversible anesthetic in *Hydra*

**DOI:** 10.1371/journal.pone.0224221

**Published:** 2019-10-24

**Authors:** Tapan Goel, Rui Wang, Sara Martin, Elizabeth Lanphear, Eva-Maria S. Collins

**Affiliations:** 1 Department of Physics, University of California San Diego, La Jolla, CA, United States of America; 2 Department of Biology, Swarthmore College, Swarthmore, PA, United States of America; 3 Department of Bioengineering, University of California San Diego, La Jolla, CA, United States of America; Imperial College London, UNITED KINGDOM

## Abstract

The ability to make transgenic *Hydra* lines has allowed for quantitative *in vivo* studies of *Hydra* regeneration and physiology. These studies commonly include excision, grafting and transplantation experiments along with high-resolution imaging of live animals, which can be challenging due to the animal’s response to touch and light stimuli. While various anesthetics have been used in *Hydra* studies, they tend to be toxic over the course of a few hours or their long-term effects on animal health are unknown. Here, we show that the monoterpenoid alcohol linalool is a useful anesthetic for *Hydra*. Linalool is easy to use, non-toxic, fast acting, and reversible. It has no detectable long-term effects on cell viability or cell proliferation. We demonstrate that the same animal can be immobilized in linalool multiple times at intervals of several hours for repeated imaging over 2–3 days. This uniquely allows for *in vivo* imaging of dynamic processes such as head regeneration. We directly compare linalool to currently used anesthetics and show its superior performance. Linalool will be a useful tool for tissue manipulation and imaging in *Hydra* research in both research and teaching contexts.

## Introduction

Abraham Trembley’s careful and systematic studies on *Hydra* regeneration, published in his *Memoires* in 1744, brought this freshwater cnidarian into the spotlight of biological research [1]. *Hydra* is an optically transparent polyp a few millimeters in length. It consists of a hollow cylindrical body column with a head on one end, consisting of a ring of tentacles and a dome-shaped hypostome, and an adhesive basal disk on the other end. *Hydra* is composed of only a small number of cell types originating from three (ectodermal, endodermal and interstitial) stem cell lineages [2]. This anatomical simplicity, continuous cell turnover in the adult [3], and the ability to regenerate from small fragments of the body column or even from aggregates of cells [4,5] render *Hydra* a powerful system for studies of development [6], stem cell biology [7,8], and regeneration [9–12]. Furthermore, *Hydra* has a relatively simple nervous system [13,14], consisting of a few thousand cells [15] that are organized in three neuronal networks [16], making it an attractive system to study neuron development [17,18] and neuronal control of behavior [16,19].

Exploiting *Hydra*’s patterning processes and regenerative abilities via sophisticated excision and grafting studies has been a mainstay of *Hydra* research since Trembley’s original experiments. This “cut-and-paste” approach has provided fundamental insights into *Hydra* biology. For example, the excision and subsequent threading of body column rings onto fishing line allowed researchers to probe questions about oral-aboral polarization [20]. Grafting of hypostomes into body columns showed that the tip of the hypostome acts as a head organizer [21,22] long before the head organizer was biochemically analyzed [23]. Transplantation experiments were used to characterize the properties and dynamics of head inhibition [24] and estimate the length scales of head activation and inhibition [25], which helped validate the Gierer-Meinhardt model of axial patterning [26] decades before *in vivo* visualization of cells or proteins was possible in *Hydra*.

However, despite its many advantages, *Hydra* has not become a mainstream model organism due to the lack of genetic tools. This has changed in the last decade with access to a fully assembled *Hydra* genome [27], single cell RNAseq data [28], and the development of molecular tools that allow for the generation of transgenic lines [29–31]. Because of these tools, numerous recent studies have been able to address longstanding open questions that could not previously be answered. For example, the recent creation of a transgenic line expressing GCaMP6s in the interstitial lineage allowed visualization of neural activity in real time in freely behaving animals and led to the discovery of multiple discrete networks of neurons linked to specific behaviors [16]. Transgenic animals have also enabled biomechanics studies to settle key biological questions regarding the mechanism driving cell sorting during regeneration from cell aggregates [11] and the functioning of the *Hydra* mouth [32].

As *Hydra* research continues to dig deeper into such questions in the living animal, future studies will require ever more precise and repeatable manipulations, high resolution live imaging, or a combination thereof to fully exploit transgenic strains and other new technologies. Because *Hydra* is in a continuous dynamic state of extension-contraction and responds rapidly to stimuli such as touch and light [33], a reversible way of slowing or preventing the animal’s movements would greatly facilitate these kinds of experiments. The search for a reliable and reversible relaxant in *Hydra* has driven the field to explore an array of compounds, with the most prominent being urethane [34–38], heptanol [39,40], and chloretone [41–44]. Urethane and heptanol have broad effects on *Hydra*. Urethane reverses the transepithelial potential, causing adverse effects upon several hours of exposure [34]. Heptanol blocks epithelial gap junction communication in the body column [45]. Chloretone is reportedly nervous-system specific, but *Hydra* was observed to develop tolerance to the anesthetic within hours of exposure [44]. Thus, existing anesthetics have limitations and there is an urgent need for an alternative that reliably immobilizes *Hydra* without causing tolerance or adverse health effects.

Here, we report on linalool as a novel, safe and fully reversible anesthetic for *Hydra*. Linalool is a monoterpenoid alcohol found in flowers and frequently used in cosmetic products [46]. It has been shown to have anesthetic or sedative activity in mice [47], catfish [48] and flatworms ms [49]. Linalool exists in two enantiomeric for ms with different pharmacological effects. In humans, the (S)-enantiomer causes an increase in heart rate while the (R)-enantiomer works as a stress relieving agent [50]. In contrast, in catfish the (S)- enantiomer acts as a sedative [48]. Here, we demonstrate that a racemic mixture of linalool enables live imaging of *Hydra*, including the acquisition of fluorescence time-lapse movies and multichannel z-stacks at high magnification. Linalool is fast acting–a 1 mM solution of linalool anesthetizes an animal within 10 min of exposure, with recovery occurring in approximately the same time after removal from the solution. Because anesthesia using linalool is reversible, the same animal can be imaged consecutively over the course of days, enabling dynamic studies of long-term processes such as head regeneration and budding. Furthermore, linalool facilitates the rapid execution of precise tissue manipulations such as tissue excisions and grafting. Linalool has been reported to be a cytostatic agent in cancer cells *in vitro* [51]; therefore, we also investigated this possibility in *Hydra*. We found no significant effects of prolonged (3-day) continuous linalool exposure on budding rates, mitotic activity, or cell viability. In contrast, 3-day continuous exposure to linalool partially suppressed regeneration in amputated animals, but regeneration could be rescued by removal of the anesthetic. Thus, linalool may also be a useful tool for manipulating regeneration dynamics. In conclusion, we find that linalool outperforms other currently used anesthetics and enables *in vivo* manipulations and live imaging of *Hydra* with precision and ease of use.

## Results

### Linalool is a fast acting and reversible anesthetic

**Fig 1:**
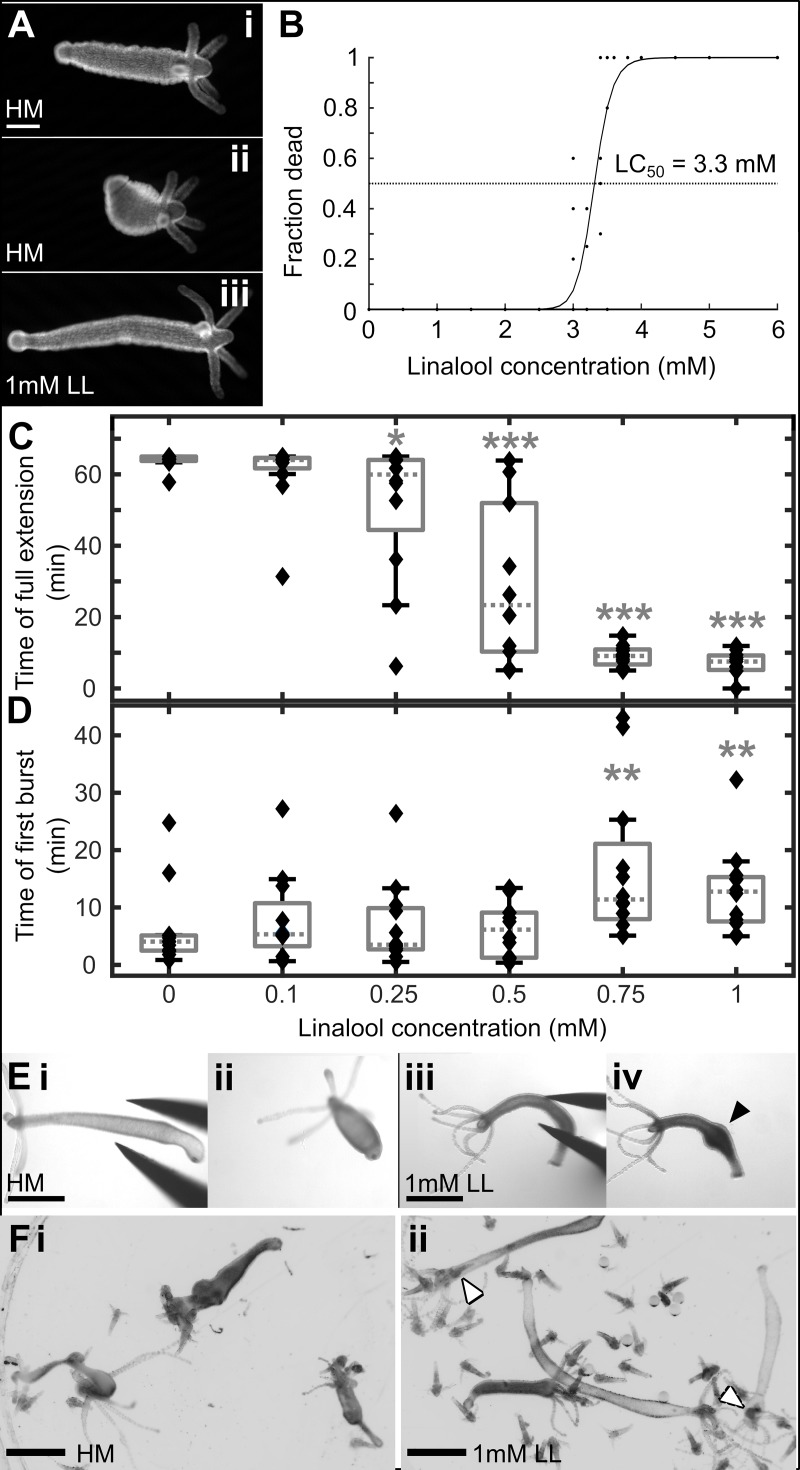
Linalool as an anesthetic. A. Representative images of *Hydra* polyps before (i. extended, ii. contracted) and after (iii) incubation in 1 mM linalool (abbreviated to LL). Scale bar: 200 μm. B. 3 hr incubation in linalool concentrations exceeding 3 mM causes lethality. Each point represents a single technical replicate containing 8–10 animals. C. Box plot showing time of full extension after last observed contraction burst during 65 min incubation in linalool concentrations up to 1 mM. 1mM linalool takes 7.53 min (5.44, 9.03) (median (25^th^ percentile, 75^th^ percentile)) to anesthetize the animals. (*), (**) and (***) indicate statistically significant difference from 0 mM linalool at p<0.05, p<0.01 and p<0.001 respectively (Mann-Whitney U test). Data from 3 technical replicates containing 3–4 animals each for every concentration. Each data point corresponds to one animal. D. Box plot showing time of first observed contraction burst during 120 min recovery in HM following 65 min of anesthesia in linalool. Animals recover in 12.77 min (7.72, 15.13) (median (25^th^ percentile, 75^th^ percentile)) after incubation in 1mM linalool. (*), (**) and (***) indicate statistically significant difference from 0 mM linalool at p<0.05, p<0.01 and p<0.001 respectively (Mann-Whitney U test). Data from 3 technical replicates containing 4 animals each for every concentration. Each data point corresponds to one animal. E. Pinch response. i. *Hydra* polyp in HM. ii. Polyp in HM shows a global body column contraction in response to pinching. iii. *Hydra* polyp incubated in 1 mM linalool for 10 min. iv. Anesthetized polyp shows only local swelling after pinch, indicated by black arrowhead. Images representative of n = 5 animals per replicate in 2 technical replicates. F. 30 min feeding response in 4-day starved polyp. i. *Hydra* polyps in HM readily capture and ingest *Artemia* (brine shrimp), with multiple *Artemia* clearly visible within the body column of each animal. ii. *Hydra* polyps incubated in linalool for 10 min prior to introduction of *Artemia* have a strongly reduced reaction, and only rarely ingest *Artemia*. White arrowheads indicate *Artemia* inside polyps. Several animals have not ingested prey at all, and those that have contain a maximum of one *Artemia* each. Scale bars for E, F: 1 mm.

Intact polyps in *Hydra* Medium (HM) continuously exhibit body shape changes such as contractions, extensions, bending, as well as tentacle movements [19,52], which complicates *in vivo* manipulations and imaging. In contrast, animals incubated in 1 mM linalool (LL) for 10 min appear relaxed, with tentacles splayed out and the mouth assuming a conical shape (Fig 1A).

We investigated the effect of various linalool concentrations on animal health within 3 hours of incubation (Fig 1B) and found that concentrations ≥ 2 mM caused negative health effects on the animals, such as an abnormal body shape, contracted tentacles, and partial disintegration (S1 Fig). Death was observed at concentrations of 3 mM and beyond, following the 3h exposure. We determined the LC_50_ to be 3.31 mM (95% confidence interval 3.27 mM to 3.36 mM) using the same approach as in [53]. We then empirically determined the optimal working concentration for linalool by measuring and comparing induction and recovery times for different sublethal concentrations.

No negative health effects were observed at or below 1mM linalool. Induction time of anesthesia decreased with increasing concentration of linalool to about 10 min at 1mM (Fig 1C), while recovery time remained between 10–20 min for all concentrations tested (Fig 1D). After a 1 h incubation in 1 mM linalool, polyps regained their spontaneous contractions in about 13.0 (8.8, 17.2) min (mean (95% confidence interval), n = 12 across 3 technical replicates) (Fig 1D). Therefore, we determined that the highest tolerated dose, 1 mM, was the best concentration to use in experiments.

Polyps incubated in 1 mM linalool for 10 min no longer exhibit the “pinch response”, a global longitudinal contraction that is observed upon gently squeezing the body column of a polyp in HM with forceps (Fig 1Ei and Fig 1Eii, S1 Movie). Polyps in 1 mM linalool swelled at the site of pinching but did not contract globally (Fig 1Eiii and Fig 1Eiv). However, upon being returned to HM, the polyps regained their response to pinching within 5 min (n = 18, across 3 replicates, S2A Fig). Taken together, these results demonstrate that linalool prevents both spontaneous and mechanically induced contractions in *Hydra*.

Mechanically induced body column contractions are known to be mediated by the ectodermal epithelial layer, and epithelial (nerve-free) animals in HM retain their pinch response despite lacking spontaneous contraction behaviors [45]. Therefore, to determine whether linalool affected epithelial cells directly, we tested whether nerve-free *Hydra* exhibited a pinch response in linalool. As was the case for enervated polyps, nerve-free animals lost their pinch response in linalool (S2 Movie). The loss of both spontaneous and mechanically induced contractions, in both enervated (Fig 1Eiii and Fig 1Eiv) and nerve free animals (S2 Movie) upon treatment with linalool suggests that linalool affects both the neuronal and epithelial cells.

However, 1 mM linalool does not completely paralyze the animal—we observed that some anesthetized individuals were able to capture and ingest *Artemia* (brine shrimp), although very inefficiently compared to controls (Fig 1F). We quantified the feeding response by adapting the protocol by [54]. While we found *Artemia* readily stuck to the tentacles of most animals, only 2 out of 9 animals in 1 mM linalool ingested 1 *Artemia* each in 30 min, whereas the median number of *Artemia* ingested by each animal incubated in HM was significantly higher (p = 0.00028, Mann-Whitney U test) with 13 (11, 16; 25^th^ percentile, 75^th^ percentile) for n = 9 polyps across 2 technical replicates. (S2D Fig).

The effect of linalool treatment on mechanically induced contraction and the ability to feed was rapidly reversed by moving the polyps back into HM. Following a 10 min incubation in 1 mM linalool, polyps regained the mechanically induced pinch response within 5 min of return to HM (n = 18 across 3 technical replicates; S2A Fig). The ability to capture and ingest *Artemia* was restored within 15 min of HM incubation following a 10–15 min incubation in 1 mM linalool, as quantified by the fraction of polyps that were able to capture and ingest *Artemia* at different time points after the linalool incubation (S2C Fig).

### 1 mM linalool enables precise tissue manipulations

**Fig 2:**
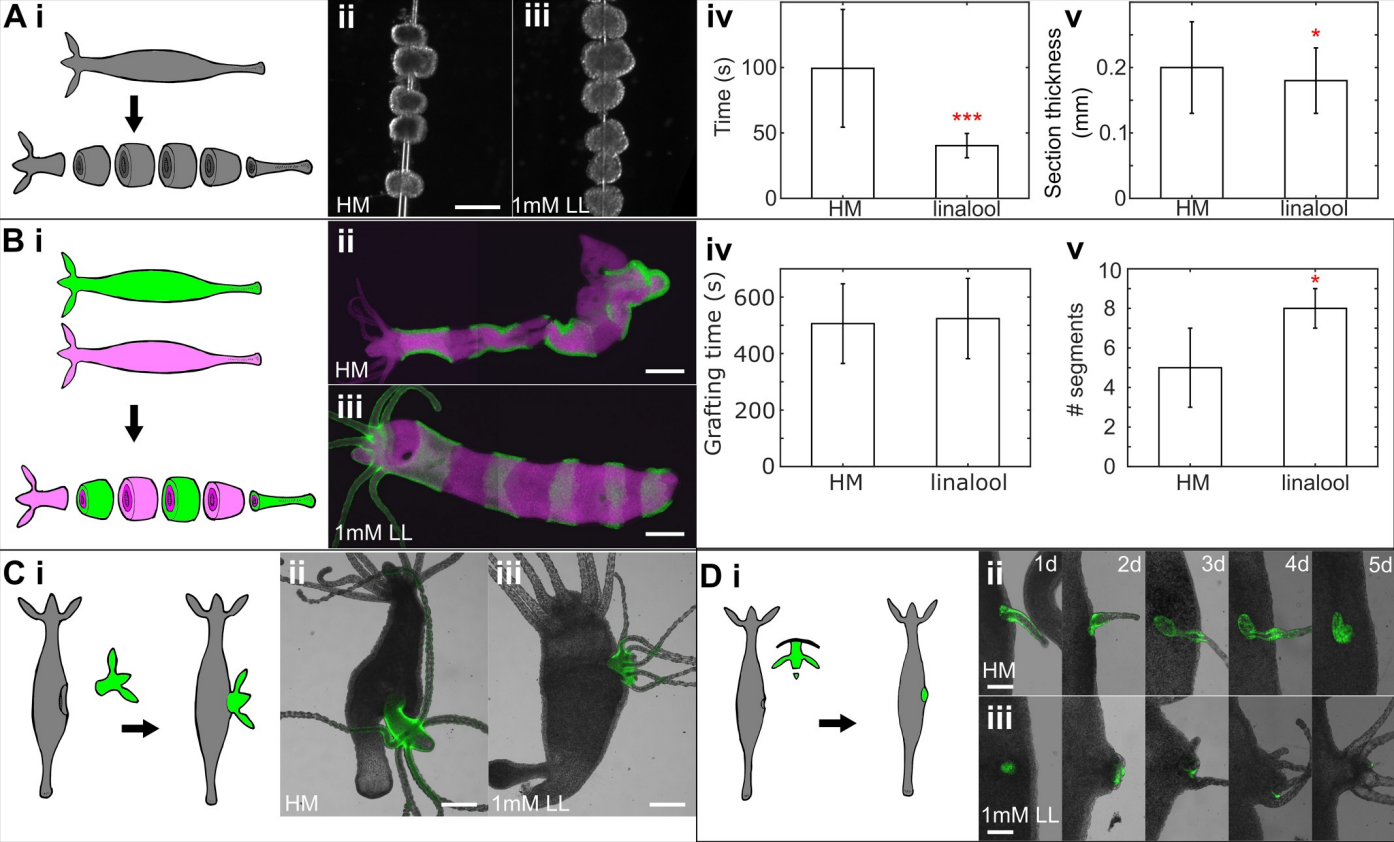
Linalool improves outcomes of surgical manipulations in *Hydra*. A. Sectioning of body column. i. Experimental schematic. ii. Sections cut in HM. iii. Sections cut in linalool. Scale bar: 400 μm iv. Time required to section a polyp in *Hydra* medium (HM) (90 ± 45 s (mean ± SD), n = 13, across 3 technical replicates) and in 1mM linalool (40 ± 9 s, n = 16, across 5 technical replicates). v. Thickness of body column sections cut in HM (0.20 ±0.07 mm (mean± SD), n = 66 sections, 15 polyps across 4 technical replicates) and in 1mM linalool (0.18 ± 0.05mm, n = 99 sections, 19 polyps across 6 technical replicates). Error bars represent SDs. (*), (**) and (***) indicate statistical significance at p < 0.05, p < 0.01 and p < 0.001 respectively, calculated using a 2 tailed t-test. B. “Zebra grafting”. i. Experimental schematic. ii. Representative animal grafted and healed in HM. iii. Representative animal grafted and healed in linalool. Scale bars: 400 μm. All grafts are shown in S3 Fig. iv. Time taken to assemble grafts in HM (506 ± 141 s (mean ± SD)) and in 1 mM linalool (524 ± 142) (n = 8, across 2 technical replicates each for HM and 1 mM linalool). v. Number of segments in completed graft in HM (5 ± 2 (mean ± SD)) and in 1 mM linalool (8 ± 1) (n = 8, across 2 technical replicates each for HM and 1 mM linalool). Error bars represent SDs. (*) indicates statistically significant difference from grafts in HM at p < 0.05 (2-tailed t-test). C. Head transplantation into gastric region. i. Experimental schematic. ii. Representative animal grafted and healed in HM. iii. Representative animal grafted and healed in 1mM linalool. Scale bars: 400 μm. D. Head organizer transplantation into gastric region. i. Experimental schematic. ii. Animal grafted in HM imaged daily over 5 days. iii. Animal grafted in 1 mM linalool imaged daily over 5 days. Scale bars: 200 μm. Linalool did not improve hypostome cutting times, which were 60 s (50, 69) (median, (25^th^ quartile, 75^th^ quartile), measured for n = 17 grafts) in HM and 50 s, (38, 66) (n = 17) in linalool, but slightly improved success of the induction of ectopic axes (6/25 in HM versus 11/25 in linalool) and significantly shortened grafting time to 134 s (104, 209) (n = 17) compared to 196 s (147, 258) (n = 17) in HM.

Recent studies have shown that the regeneration outcome in *Hydra* could be influenced by the geometry of tissue pieces excised from the body column [55]. Making precise cuts is also useful for manual sections of the body column for use in immunohistochemistry and histology. To test whether linalool allowed for improved precision of cuts and thus would be a useful tool for such studies, we compared the excision of tissue rings from animals incubated in HM with those incubated in 1 mM linalool. When sectioning animals to obtain pieces of body column tissue, the application of linalool did not drastically improve the average thickness of the sections (Fig 2Av), but significantly reduced the time required to section the animals from 99 ± 45 s (mean ± standard deviation (SD), n = 13 across 3 technical replicates) per animal in HM to 40 ± 9 s (mean ± SD, n = 16 across 5 technical replicates) per animal in 1 mM linalool (p = 0.00002, 2-tailed t-test) (Fig 2Aiv). The reductions in variability and in average time are due to the suppression of the animal’s natural contractile response to touch, removing the need to wait for the polyp to extend following each cut.

The improvements possible using linalool become more readily apparent in grafting experiments. A “zebra graft” to create a chimeric animal consisting of bands of differently labeled tissue produced a significantly better result when linalool was employed (Fig 2B). While the time to assemble the grafts was comparable with or without linalool treatment (Fig 2Biv), the average number of segments per graft was significantly higher (p = 0.03, 2-tailed t-test) for grafts in linalool (8 ± 1 segments per graft, n = 8 grafts) than those made in HM (5 ± 2 segments per graft, n = 8 grafts; Fig 2Bv). This difference is due to a combination of two effects. First, the animals do not move in linalool and second, they are extended. Thus, they are more quickly and easily cut into smaller segments, which are in turn easier to thread onto a needle. Morphology of grafts made in HM was also more frequently abnormal compared to those made in linalool (S3 Fig). The observed morphological abnormalities are likely due to tissue movement during healing, causing the cut edges of the pieces to become misaligned while on the needle, thus preventing the segments from healing smoothly together as described previously [56]. A similar effect was observed when grafting heads onto body columns (n = 3 per condition), following the procedure described in [57]. Linalool allowed more precise decapitation of the donor animal, reducing the amount of extraneous body column tissue, and guaranteed better positioning of the graft on the recipient animal. Grafts carried out in HM tend to have the donor head protruding at an angle, again due to misalignment of the cut surfaces during healing (Fig 2C).

Finally, linalool is beneficial in hypostome grafts, carried out as previously described [58]. We conducted 25 hypostome grafts each in HM and in 1 mM linalool and scored at 4 days after grafting for retention of donor tissue and for formation of an ectopic body axis from recipient tissue with donor tissue limited to a small part of the new head (Fig 2Diii), as in [21,58]. Grafts that retained donor tissue but failed to induce an axis fell into several broad categories: donor tissue that either failed to form any structure or induced only a tentacle before being resorbed (Fig 2Dii), or donor tissue that formed the entirety of an ectopic head with no host tissue involvement (S4 Fig). We found a slight but statistically non-significant improvement in the number of grafts that induce an ectopic axis when linalool is used (6/25 in HM vs. 11/25 in linalool). For two technical replicates containing n = 16 grafts per condition, we individually recorded the time taken to excise the donor hypostome and to conduct the graft. Linalool did not significantly improve the time required to cut hypostomes (p = 0.1221, Mann-Whitney U test), but grafting times were significantly shorter (p = 0.0226, Mann-Whitney U test) in linalool, with 134 s (104, 209; median, (25^th^ quartile, 75^th^ quartile)), compared to in HM with 196s (147, 258).

### Incubation in 1 mM linalool enables high quality short-term fluorescence imaging

To test whether the immobilization in 1 mM linalool was sufficient to allow for *in vivo* fluorescence imaging, we imaged animals incubated in 1mM linalool under various conditions and compared the results to those obtained from imaging animals in HM.

**Fig 3:**
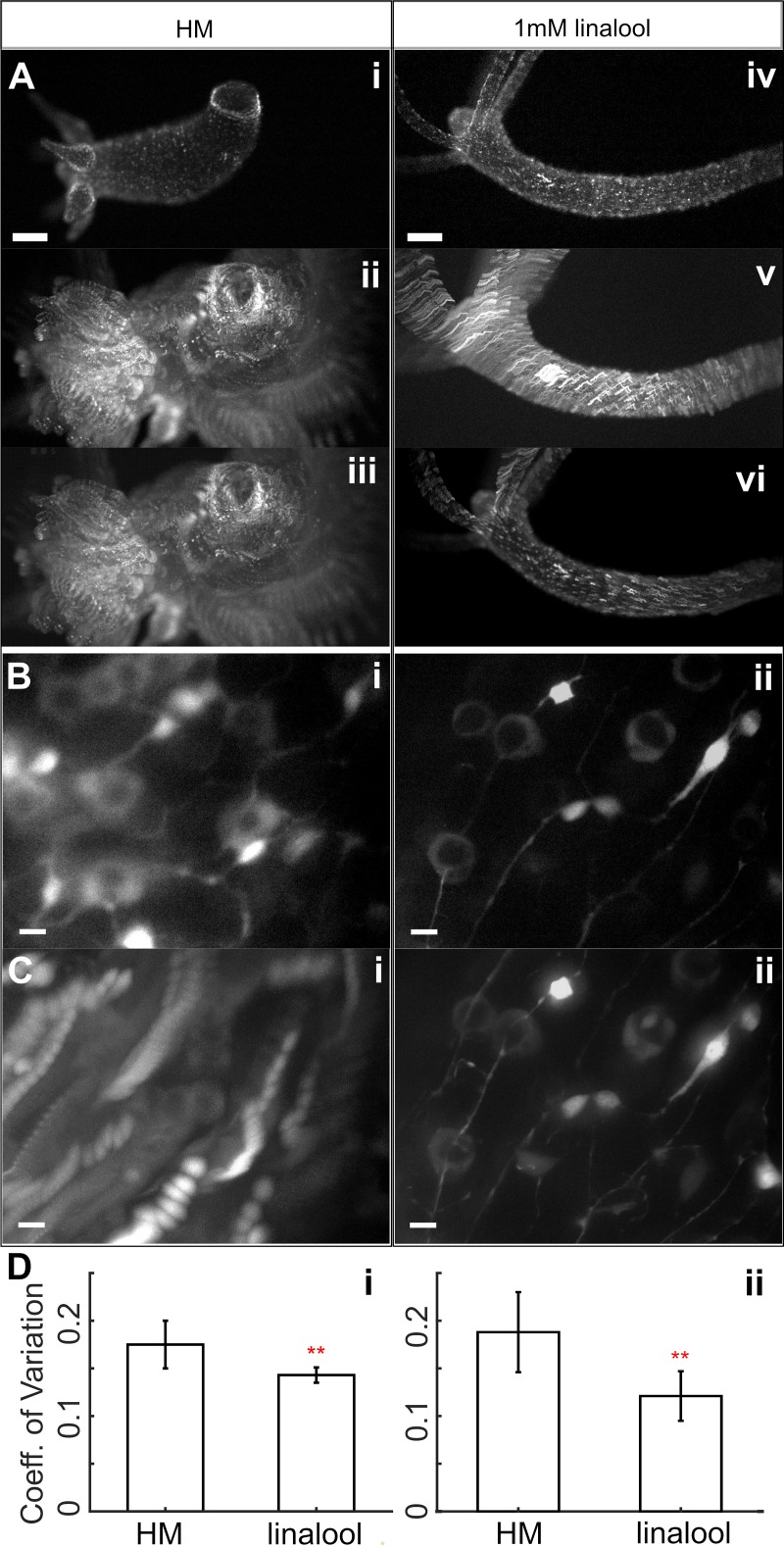
Single channel live imaging in linalool. A. Unconstrained GCaMP6s *Hydra* imaged at low magnification. i. single image in HM. ii. Maximum intensity t-projection of a 10 s video in HM. iii. Rigid body correction of HM video projection. iv. Single image in 1 mM linalool. v. Maximum intensity t-projection of 10 s video in linalool. vi. Rigid body correction of linalool video projection. Scale bars: 200 μm. B. Single slice from a 7.5 μm thick z-stack of a GCaMP6s animal imaged at 60x magnification with a resolution of 0.25 μm along the z-axis at a 500 ms exposure per slice using blue excitation in (i) HM and (ii) 1 mM linalool. C. Maximum intensity projection of high magnification z-stacks in (i) HM and (ii) 1 mM linalool. Scale bars: 10 μm. D. Coefficient of variation for (i) low magnification imaging in HM (0.175 ± 0.025 (mean ± SD)) and linalool (0.143 ± 0.008) calculated from n = 10 polyps across 2 technical replicates (ii) high magnification imaging in HM (0.188 ± 0.042 (mean ± SD)) and linalool (0.121 ± 0.026) calculated from n = 6 polyps across 2 technical replicates. Error bars represent SDs. (**) indicates statistically significant difference at p < 0.01 as determined by a 2 tailed t-test.

First, we used single channel fluorescence imaging using polyps expressing GCaMP6s in the interstitial cell lineage [16], because this transgenic line allows for the visualization of individual neurons and subcellular processes such as dendrites. We imaged unconstrained animals at low magnification (Fig 3A, S3 Movie). Unconstrained animals in HM moved significantly during the 10s acquisition, as shown by a maximum intensity projection of the time series (Fig 3Aii). In contrast, polyps incubated in 1 mM linalool for at least 10 min only exhibited drift (Fig 3Aiv and 3Av), which can be corrected for with standard post-processing methods (Fig 3Avi), whereas these methods do not correct for the motion observed in the control, because the animal exhibits non-linear body shape changes (Fig 3Aiii).

We also acquired 7.5 μm thick z-stacks of the body columns of intact polyps mounted in tunnel slides [32] at high magnification (Fig 3B and 3C). The image quality of individual slices was better when imaging anesthetized animals (Fig 3B), but the difference in stability and thus image quality becomes most evident when comparing maximum intensity projections of the entire z-stack (Fig 3C). The animals in linalool were sufficiently still to allow the resolution of subcellular features such as neuronal processes, whereas the animals in HM moved too much, making z-stacks impractical (Fig 3C and S4 Movie). As the tissue stretched and compressed anisotropically during those movements, it was not possible to correct this motion through post-processing. We quantified the motion under these two imaging conditions (HM, 1 mM linalool) using the coefficient of variation (see Methods). As expected from the images (Fig 3), the coefficient of variation was significantly higher for image sequences acquired in HM than for those acquired in 1 mM linalool (S1 Table, Fig 3D).

**Fig 4:**
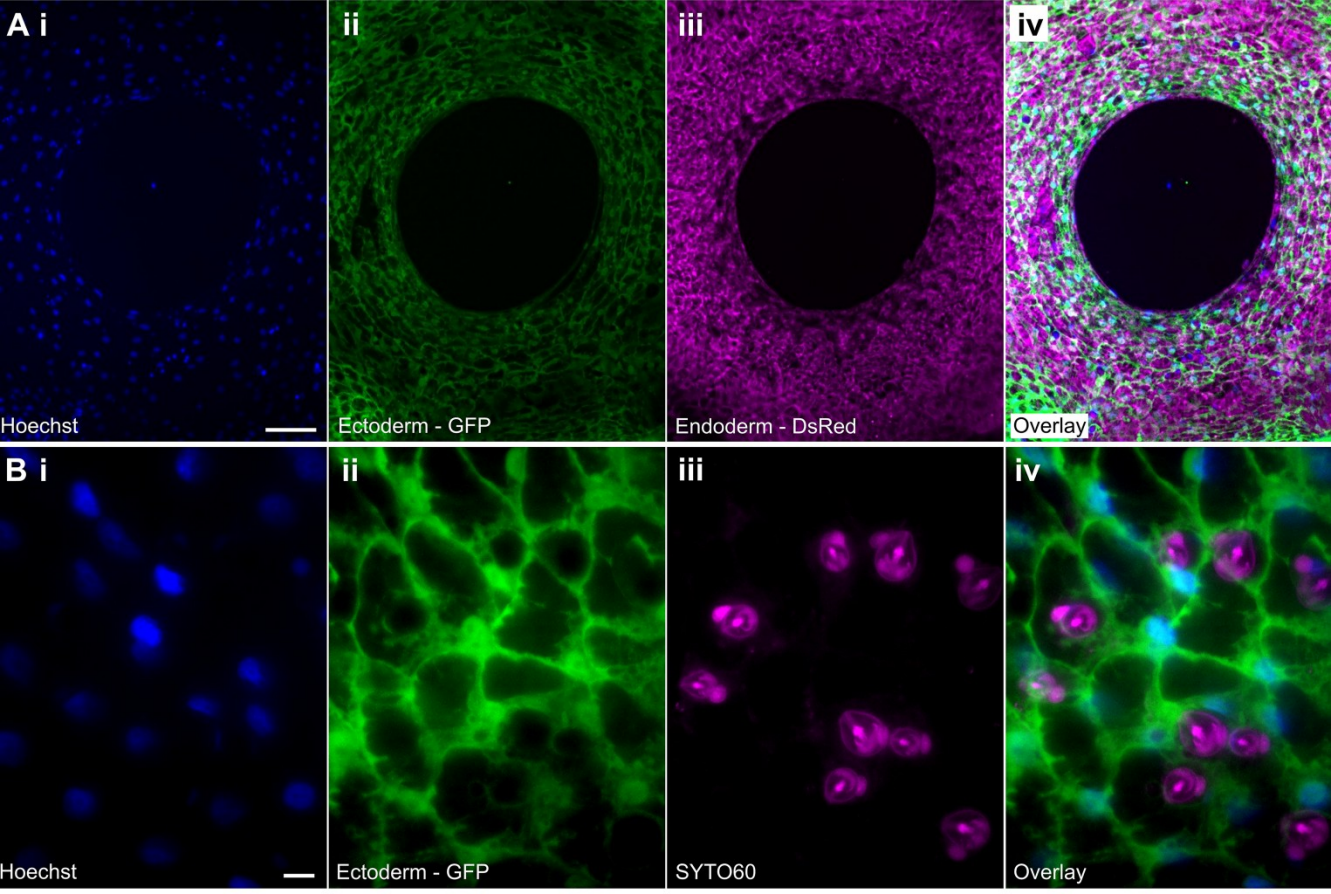
Linalool enables high resolution imaging in multiple channels. A. Low magnification maximum intensity projection of a z-stack acquired of an open *Hydra* mouth in 1mM linalool using i. Hoechst 33342, ii. Ectoderm—GFP, iii. Endoderm–DsRed2, iv. overlay. 5 μm slice thickness, 6 slices total. Scale bar: 100 μm. B. High magnification maximum intensity projection of a z-stack of the body column tissue acquired in 1mM linalool using i. Hoechst 33342, ii. Ectoderm–GFP, iii. Nematocysts–SYTO 60, iv. overlay. 0.25 μm z-step, 17 slices total. Scale bar: 10 μm. The reduced animal motion allows for acquisition of multiple z-slices in 3 channels.

Next, we tested the performance of 10 min incubation in 1mM linalool for the acquisition of multi-channel z-stacks at low (10x) and high magnification (60x). Control videos in HM were not attempted due to the unsatisfactory results obtained in single channel imaging as described in the preceding paragraphs (Fig 3). By exposing animals to 1mM linalool in the presence of 2mM reduced glutathione, we were able to induce mouth opening (Fig 4A). The animal is sufficiently still to allow for simultaneous visualization of nuclei positions and cell boundaries using different excitation wavelengths at 10x magnification. We also took 3-channel time-lapse movies of heads exposed to reduced glutathione below the activation threshold for opening to illustrate the overall stability that can be achieved using linalool, allowing for co-localization studies of dynamic processes (S5 Movie).

Furthermore, we used mouth opening to test if calcium imaging was possible in linalool-treated animals. Epithelial GCaMP animals [58] incubated in 1mM linalool for 10 min opened their mouth in response to 2mM reduced glutathione and calcium waves could be observed (S6 Movie). We also observed local calcium signaling in the body column in response to pinching with tweezers (S8 Movie). Together, these data demonstrate that linalool does not interfere with epithelial calcium signaling and that behaviors that are not suppressed by linalool can be studied using GCaMP animals.

Finally, we tested whether animals were sufficiently immobile to obtain high quality z-stacks at high magnification (60x) in multiple channels (UV, blue, and green excitation; Fig 4B). Notably, when testing live dyes for this purpose, we found that the SYTO 60 red fluorescent nucleic acid stain is specific to nematocysts of all types in *Hydra* (Fig 4Biii), determined by comparing morphology of stained structures to previous descriptions of nematocyst types [59]. Thus, SYTO 60 is a useful tool for studying nematocysts *in vivo*.

While motion was not completely suppressed in 1 mM linalool and extended exposure to short wavelength light caused the animal to escape the field of view, it was nevertheless possible to achieve high quality multichannel imaging (Fig 4B). Thus, linalool is a useful tool for *in vivo* co-localization studies at high magnification, which are impossible to perform in HM.

### Linalool allows for repeated short-term fluorescence imaging

**Fig 5:**
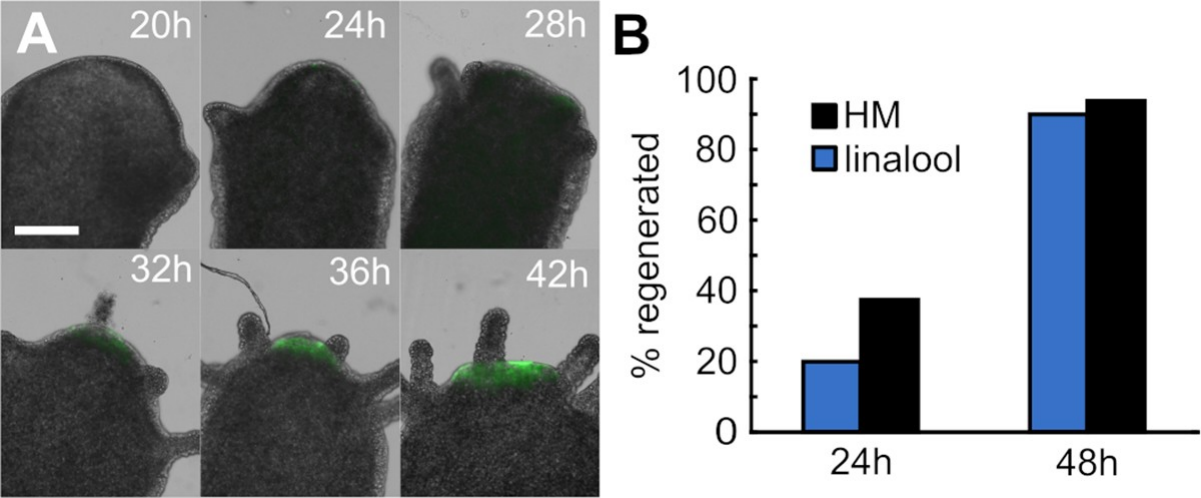
Linalool enables repeated high-resolution imaging. A. Head regeneration in a transgenic HyBra2 promoter::GFP polyp imaged at high resolution every 4 h from 12 h to 48 h. Subset of images shown. Scale bar: 0.5 mm B. Repeated anesthesia and recovery do not impact regeneration speed or outcome (n = 10 animals HM, n = 16 animals linalool, 3 technical replicates). Differences between conditions not statistically significant at p = 0.05 level (Fisher’s Exact test).

A major strength of linalool as a reversible anesthetic is the ability to repeatedly anesthetize and image the same animal over the course of days, thus allowing the acquisition of dynamic data of cellular processes in a single animal. To illustrate this capability, we decapitated transgenic HyBra2 promoter::GFP animals and allowed them to regenerate in HM. We imaged head regeneration over the course of 2 days, using repeated short-term 15 min incubations in 1 mM linalool to acquire a total of 11 high resolution images of the same animal (Fig 5A). When not being imaged, the regenerating animals were returned to HM. In this way we were able to observe the development of the hypostome and tentacles and also observe a gradual increase in GFP signal beginning at 24 h. The same technique of repeated linalool exposure was used to image the tissue grafts in Fig 2.

We also confirmed that the timing and outcome of head regeneration in animals repeatedly anesthetized for imaging did not significantly differ from that observed in untreated controls (Fig 5B). Thus, linalool is a valuable tool for repeated live imaging applications, which will be useful to study long term processes, such as regeneration and budding.

### Long-term effects of linalool

**Fig 6:**
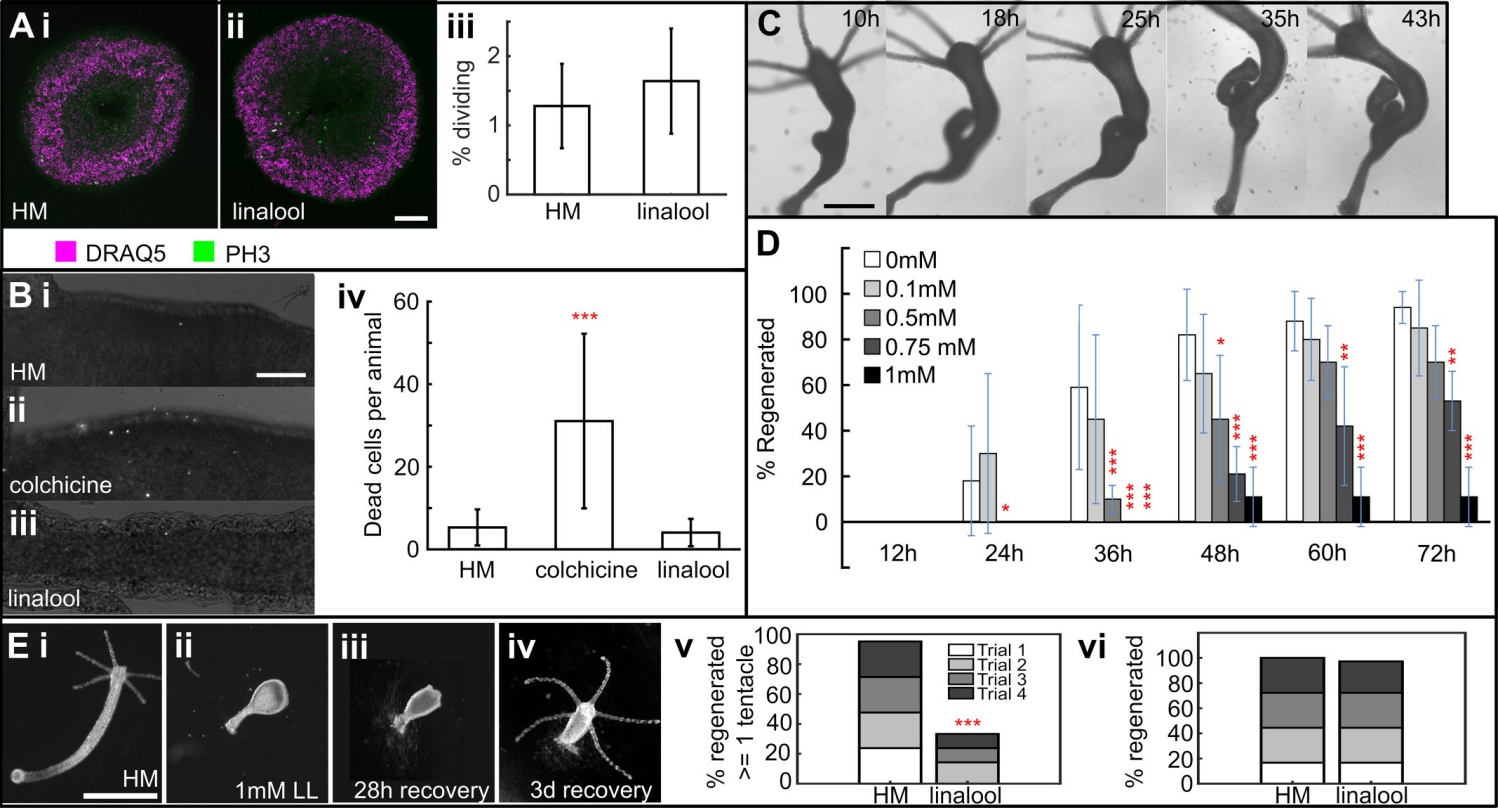
Effect of long-term continuous linalool exposure. A. 3-day incubation in 1 mM linalool does not impact rate of cell division. Slices stained with DRAQ5 (nuclei) and anti-PH3 (phospho-histone H3, dividing cells). i. Representative image of body column sections from polyps incubated 3 days in HM. ii. Representative slice from polyps incubated 3 days in 1 mM linalool. iii. Percentage of dividing cells in animals incubated 3 d in HM or 1mM linalool. Mean ± SD: HM = 1.3 ± 0.6, linalool = 1.6 ± 0.8. n = 18 across 5 technical replicates. Difference not statistically significant at p < 0.05 (2-tailed t-test). Error bars represent SD. Scale bar: 100 μm. B. 3-day incubation in 1 mM linalool does not damage or kill cells. Representative images of polyps stained with propidium iodide after incubating for i. 3 days in HM, ii. 24 h in 0.04% colchicine, and iii. 3 days in 1 mM linalool. iv. Mean number of dead cells per animal after incubation in HM (5 ± 4, n = 38), in colchicine (31 ± 21, n = 28) and linalool (4 ± 3, n = 39). Error bars represent SD. (***) indicates statistically significant difference from linalool at p < 0.001 (2-tailed t-test). Scale bar: 100 μm. C. Long term incubation in linalool does not impact budding. Representative images of a budding polyp continuously incubated and imaged in 1 mM linalool. Scale bar: 500 μm. D. Long term incubation in linalool prevents head regeneration. Error bars represent SD (0 mM n = 17, 0.1 mM n = 20, 0.5 mM n = 40, 0.75 mM n = 19, 1 mM n = 19; 3 technical replicates). (*), (**) and (***) indicate statistically significant difference from 0 mM at p<0.05, p<0.01 and p<0.001 respectively (Fisher’s Exact Test). E. Recovery in HM rescues the head regeneration defect. i. Polyp incubated in HM for 68 h after decapitation. ii. Polyp incubated in 1 mM linalool for 68 h after decapitation. iii. Decapitated polyp recovered for 28 h after 3 d in 1mM linalool, iv. Polyps recovered for 3 d after 3 d in 1mM linalool. Scale bar: 1 mm. v. Head regeneration is suppressed by incubation for 3 d in 1 mM linalool. Only 14/42 polyps incubated in 1 mM linalool regenerated at least one tentacle at the end of 3 d incubation compared to 40/42 polyps in HM (across 4 technical replicates). (***) denotes that the difference is statistically significant at p < 0.001 (Fisher’s Exact test) when comparing overall numbers. vi. Head regeneration is rescued in linalool- incubated animals after 3 d recovery in HM. 35/36 polyps incubated in 1 mM linalool regenerated heads at the end of 3 d recovery compared to 36/36 polyps in HM, across 4 technical replicates. The difference is not statistically significant at p < 0.05 (Fisher’s Exact test) when comparing overall numbers.

Due to the reported cytostatic effect of linalool on cancer cells in culture [51], we investigated whether linalool has similar effects in *Hydra*. The cell cycle lengths in interstitial and epithelial cells are approximately 1 [60] and 3 days [61], respectively. Therefore, we continuously incubated intact polyps for 3 days in 1 mM linalool, exchanging the solution every 24 hours to account for volatility. We did not observe significant changes in the mitotic index (Fig 6A) nor in the rate of cell death (Fig 6B) in the body column of intact polyps. Furthermore, budding seemed to occur normally, as verified using 3-day continuous time-lapse imaging (Fig 6C, S5 Fig).

Based on these results, we attempted to image head regeneration using continuous incubation in 1 mM linalool. Continuous incubation would be advantageous compared to consecutive mounting and imaging sessions as it would minimize interaction with the sample and could be fully automated. We found that decapitated *Hydra* experienced a significant delay in head regeneration when continuously exposed to 1 mM linalool over the course of 3 days. Anesthetized body columns were observed to shed cells and assume a lollipop shape (Fig 6Ei and 6Eii), and a few animals disintegrated completely. A third of the animals (14/42 across 4 technical replicates) were less affected and showed 1–2 small tentacle buds at the end of 3 days (S6 Fig). If removed from linalool after 3 d, however, the remaining two thirds of the animals, which showed no visible signs of regeneration, recovered. Tentacle buds were observed as early as 1d into recovery and all polyps had fully regenerated their heads after 3d of recovery (Fig 6E). Foot regeneration was similarly suppressed in 3-day continuous 1 mM linalool exposure and was also rescued after the animals were moved into HM (S7 Fig). This suggests that the effects of linalool on regeneration are not specific to the head.

The observed head regeneration delay in continuous linalool exposure was observed for concentrations as low as 0.5 mM for up to 48 h (Fig 6D), and at 0.75 mM, 50% of the animals did not regenerate heads within 3 days. However, since even 1 mM linalool was ineffective in sufficiently immobilizing animals to allow for long-term imaging with cellular resolution (S7 Movie), these lower concentrations are not viable alternatives.

Finally, we tested whether the inhibition of regeneration is caused by an effect on the nervous system, as it had previously been suggested that the nervous system plays a role in head regeneration [62]. To this end, we generated nerve-free animals as described in Methods and assayed head regeneration in 1 mM linalool. Surprisingly, nerve-free animals in 1 mM linalool regenerated similarly to nerve-free animals maintained in HM. After 4 days of regeneration, 7/10 animals in HM and 4/10 in linalool showed tentacle buds across two technical replicates. By 5 days this had increased to 9/10 in HM and 6/10 in linalool (S8 Fig). There were no statistically significant differences in the fraction of head regenerates for both days (p = 0.36 for day 4 and p = 0.30 for day 5, Fisher’s Exact test). Furthermore, nerve-free animals in linalool did not assume the lollipop shape (Fig 6Eii) that we observed in enervated polyps. Together, these data suggest that linalool disrupts regeneration by perturbing the function of either neurons or other cells in the interstitial lineage.

### Comparison of linalool to other commonly used anesthetics in *Hydra* research

Whenever one introduces a new tool, it is important to compare performance with existing methods and demonstrate that the advantages of the new tool are sufficient to make its adoption worthwhile. While anesthetics were and continue to be most frequently used to relax *Hydra* prior to fixation for histological and immunohistochemistry studies [35, 36, 38, 63], the advent of modern molecular tools have brought with it an increased use for *in vivo* applications [37, 41, 42]. Table 1 provides an overview of the various anesthetics that have been reported in the literature for use in *Hydra* and examples of their respective applications.

**Table 1:** Summary of various anesthetics used to relax *Hydra*.

Chemical	Working concentration	Application	Treatment duration	Health effects	References
Urethane	2% w/v	Determination of mechanism of urethane’s action		Hyperextension; potential reversal; structural damage	[34]
	1–3% w/v	Relaxation prior to fixation	2–20 min	None reported	[35,36,38,63,64]
	2% w/v	Fluorescence microscopy	Not reported	None reported	[37]
	5*10^-2^M	Inhibition of feeding reaction			[43]
Chlorobutanol (chloretone)	0.1–0.33% w/v in bath	Reactions to chloretone exposure on 3 *Hydra* species	several hours	No apparent damage at low concentrations; habituation	[44]
	3*10^-3^M	Inhibition of feeding reaction	Not reported	None reported	[43]
	0.1% w/v	Fluorescence imaging	Not reported	None reported	[41,42]
1-Heptanol	3 mM	RNA interference	10 min at 4°C	None reported	[39]
	1% v/v	Fluorescence microscopy	Not reported	None reported	[40]
Magnesium chloride	2.5% w/v	Inhibition of feeding reaction	5 min	Extensive damage with exposure >1 hr	[32]
Menthol	Not reported	Relaxation prior to fixation	Not reported	None reported	[65,66]
	Not reported	Inhibition of feeding reaction	Not reported	Disintegration	[32]
MS-222	0.1%	Inhibition of feeding reaction	Not reported	None reported	[43]

This table is not a comprehensive summary of all *Hydra* studies that have employed anesthetics but provides an overview of examples spanning different chemicals and applications. To the best of our knowledge such a direct comparison has not previously been attempted and is thus a useful resource for the field.

**Fig 7:**
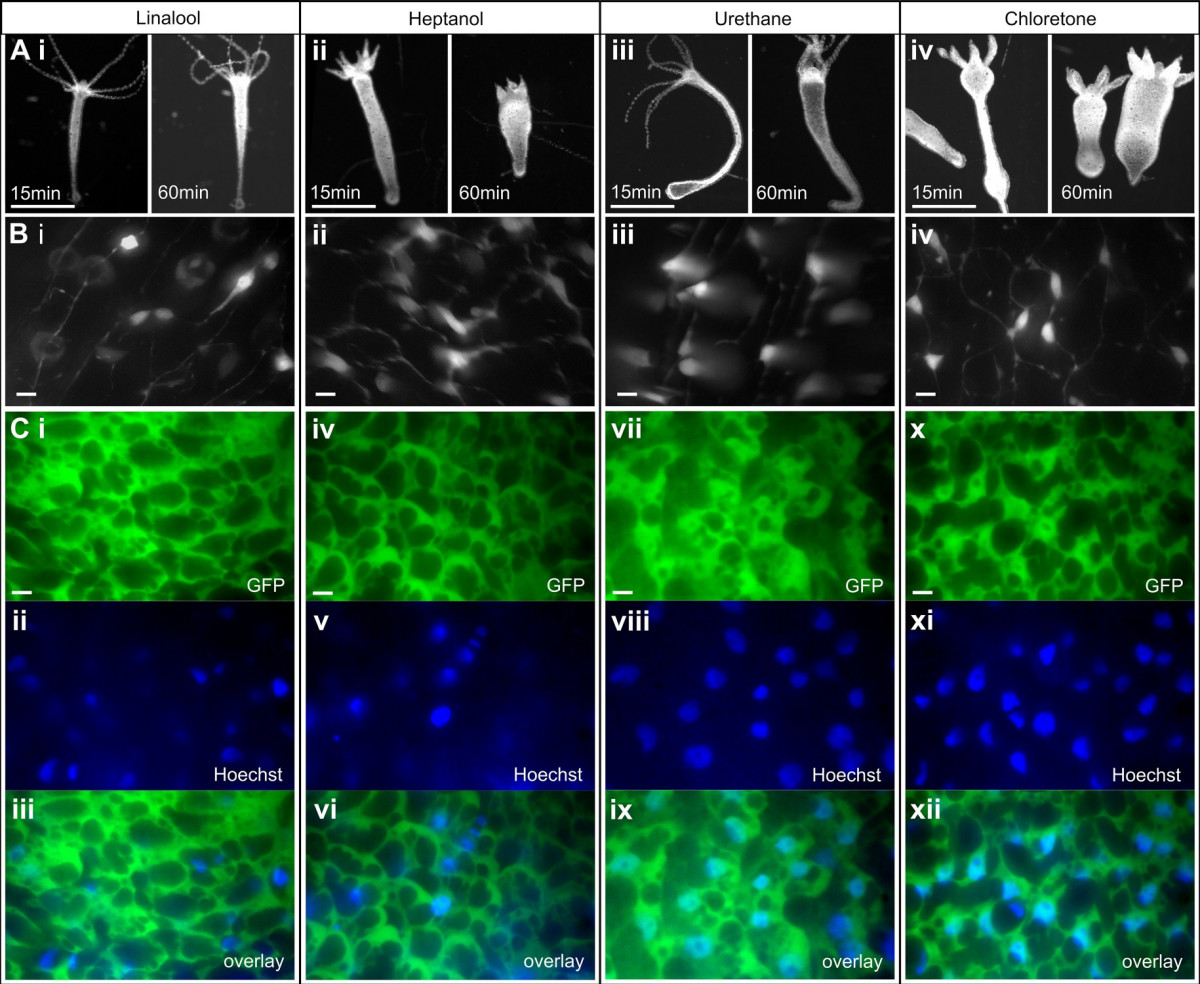
Comparison of various *Hydra* anesthetics. A. Comparisons of the same animal after 15 min and 60 min of anesthetic exposure. i. 1 mM linalool, ii. 0.04% heptanol, iii. 2% urethane, iv. 0.1% chloretone. Scale bars: 1 mm. B. Maximum intensity projections of GCaMP6s animals at 60x magnification in each anesthetic. Scale bars: 10 μm. C. Maximum intensity projections of two-channel images of watermelon animals stained with Hoechst nuclear dye at 60x magnification. GFP channel, DAPI channel, and merge (overlay) shown for each anesthetic. Scale bars: 10 μm.

**Fig 8:**
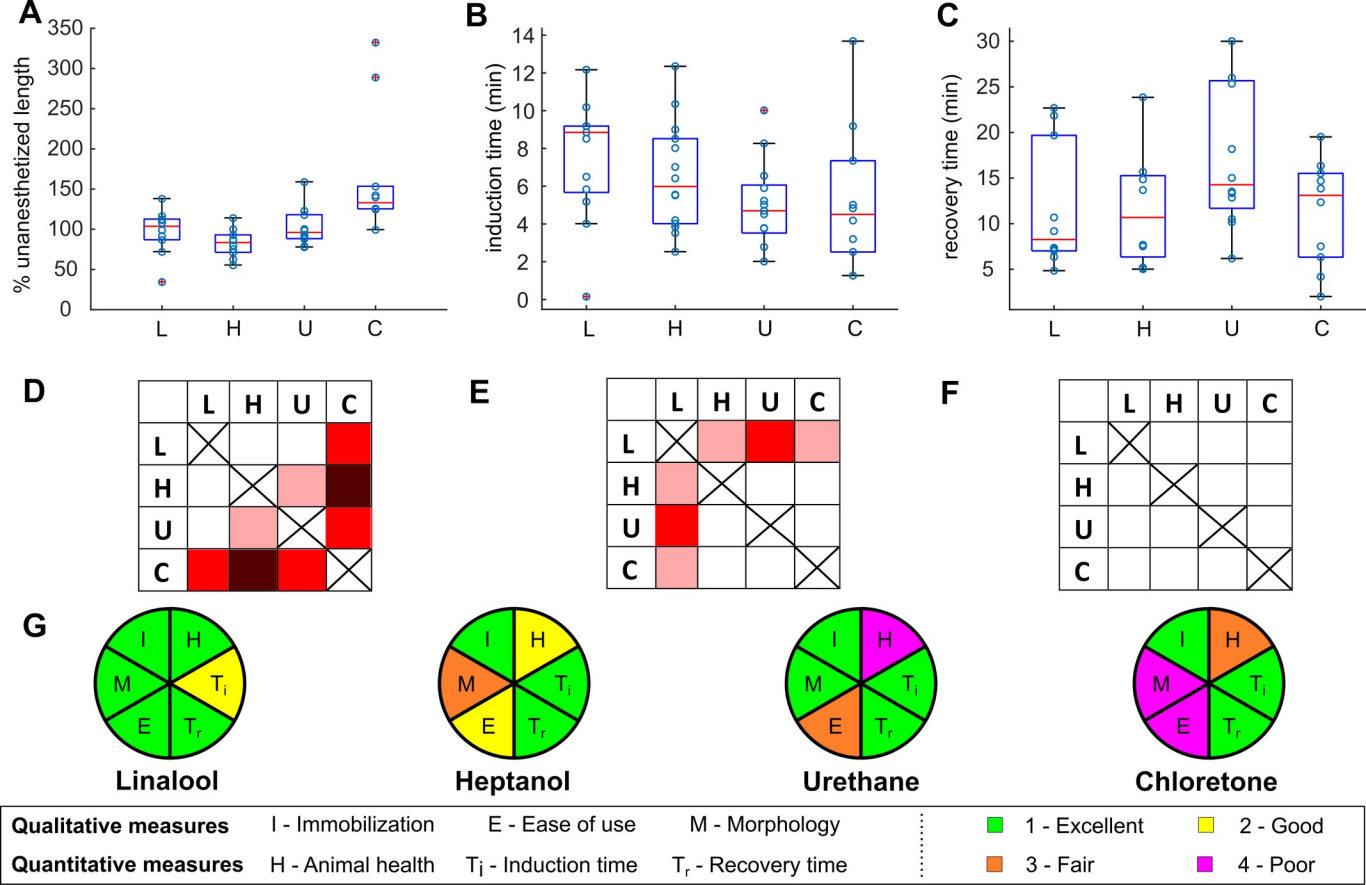
*Hydra* response to 1mM linalool (L), 0.04% heptanol (H), 2% urethane (U), and 0.1% chloretone (C). A. Percent length of anesthetized *Hydra* polyps compared to their natural state at 10min incubation. n = 10 animals per condition across 2 technical replicates. Anesthetized lengths similar to the average lengths in HM were recorded in linalool at 103% (87, 112; median (25^th^ percentile, 75^th^ percentile)), heptanol at 83% (71, 93) and urethane at 96% (88, 118), while chloretone-treated animals hyperextended at 133% (125, 153). B. Induction times across 2 technical replicates. Linalool n = 13, heptanol n = 14, urethane n = 13, chloretone n = 10. Linalool’s median induction time was 9 min (6, 9) (median (25^th^ percentile, 75^th^ percentile)) and thus significantly longer than that of heptanol at 6 min (4, 9), urethane at 5 min (4, 6) and chloretone at 5 min (3, 7). C. Recovery times across 2 technical replicates. Linalool n = 10, heptanol n = 8, urethane n = 12, chloretone n = 10. Median recovery time was 8 min (7, 17) for linalool, 11 min (7,15) for heptanol, 14 min (12, 26) for urethane and 13 min (7,15) for chloretone. (D-F) Pairwise statistical comparisons of data shown in A-C. Pink, red and dark red indicate a statistically significant difference at p<0.05, p<0.01 and p<0.001 respectively, determined using the Mann-Whitney U test between pairs of anesthetics. D. Comparison between percent length distributions. E. Comparison between induction time distributions. F. Comparison between recovery time distributions. G. Overview of the four anesthetics tested, scored on degree of immobilization, animal health following anesthesia, time to induce anesthesia, time to recover from anesthesia, morphology, and ease of use (see Methods).

Based on our literature search, the most prominent *in vivo* application of the anesthetics was fluorescence imaging using urethane, heptanol, or chloretone. We therefore compared linalool to these anesthetics. To this end we studied whether there were any differences in morphology when *Hydra* polyps are exposed to the different substances. Although we observed variability among individual polyps exposed to the same anesthetic at a fixed concentration, both in terms of morphology and in terms of immobilization speed and strength, polyps assumed characteristic shapes upon exposure to the different chemicals (Fig 7A). Following a 15 min exposure, *Hydra* polyps incubated in 1 mM linalool appear relaxed with tentacles splayed outwards and had cone-shaped hypostomes (Fig 7Ai). This morphology does not change significantly by 60 min. Animals incubated in 0.04% heptanol appear less extended at 15 min, with contracted conical tentacles. At 60 min the body columns are contracted, and the stubby tentacles persist (Fig 7Aii). Exposure to 2% urethane causes animals to extend and become very thin at 15 min, though they become swollen while remaining extended by 60 min (Fig 7Aiii). 0.1% chloretone causes initial extension without the thinness seen in urethane, followed by the formation of swellings along the body column by 15 min and contraction of both body and tentacles by 60 min (Fig 7Aiv). To quantify these differences, we calculated average body length of individual animals after 10 min incubation in anesthetic as a percentage of their average length prior to anesthesia (see Methods and Fig 8).

We found that linalool, heptanol and urethane produced similar anesthetized lengths at 10min, while chloretone showed a statistically significant increase in length at the 5% level and some hyperextended animals (Fig 8A and 8D). Because this length measure does not account for the other morphological characteristics described above (e.g. the contracted tentacles in heptanol or the formation of bumps in the body column of chloretone-treated animals) nor for the changes in morphology that were observed for heptanol and chloretone over time, we also qualitatively compared the body shapes of the anesthetized animals (Fig 7A) to those of untreated animals. Because the morphologies in linalool and urethane were the most similar to the untreated body morphology and did not change much over the course of 60 min incubation, we ranked morphology in linalool and urethane as the best, followed by heptanol (contracted body and tentacles, changing shape), and chloretone (hyperextended, contracted tentacles, bumps, changing shape).

Because most published studies specified only the concentration of anesthetic used and not the incubation time, we used concentrations that have been reported in the literature to be effective for the different anesthetics and measured induction and recovery times for direct comparison to linalool. Anesthesia in linalool is induced slower than in other anesthetics (Fig 8B and 8E). Recovery times were statistically similar between all anesthetics, with most polyps resuming normal activity within 10–20 min post-exposure (Fig 8C and 8F).

Finally, we compared the effects of long-term exposure to the different anesthetics. First, we tested a 3-day exposure to the anesthetics without changing the medium, as would be necessary for long term immobilization for continuous imaging, as in the example shown in Fig 6C for 1 mM linalool. While 1 mM linalool does not negatively affect intact polyps (Fig 6), all polyps disintegrated within 24 h upon continuous exposure to 2% urethane (S9 Fig). Under the same conditions, chloretone caused disintegration in 50% of the animals after 24 h, with most polyps disintegrating by 72 h (S9 Fig). The animals that survived the 3-day chloretone treatment without solution exchange had a fairly normal morphology and pinch response, potentially due to a developed tolerance, as previously suggested [44]. Heptanol was not lethal to *Hydra* over 3 days (S9 Fig), but as with chloretone the animals regained normal morphology and pinch response by the third day.

Subsequently we tested a 3-day incubation with media changes every 24 h, to determine whether performance could be improved by constant refreshing of the anesthetic. As seen with the previous experiment, polyps in urethane died within the first 24 h and polyps in linalool were still alive at the end of 72 h. Survival in chloretone was reduced as all polyps had died by 48 h. Similarly, only about 60% of the polyps survived in heptanol by 72 h (S9 Fig).

We also compared the performance of these various anesthetics for single and dual channel high magnification fluorescent live imaging of GCaMP6s (Fig 7B) and WM animals labeled with Hoechst (Fig 7C), respectively and did not observe a notable difference in image quality between the anesthetics.

Using these comparative data, we ranked the performance of the anesthetics in the six categories we tested (Fig 8G and Methods): ease of use, animal morphology upon exposure to anesthetic, induction and recovery times, immobilization/imaging quality, and negative health effects. As liquids, heptanol and linalool solutions are easier and quicker to prepare than urethane or chloretone, which are supplied as solids (see Methods). Morphology is closest to normal and remains stable over the course of at least 1 hour incubation in urethane and linalool (Fig 7A and Fig 8A and 8D). Induction was slightly slower in linalool compared to the other three anesthetics (Fig 8B and 8E), but recovery times after short-term exposure were similar (Fig 8C and 8F). All anesthetics tied regarding immobilization/imaging quality (Fig 7B and 7C). Linalool stands out with the least negative health effects–in contrast to the situation in the other anesthetics, not a single animal died in linalool at the end of 3-day exposures (S9 Fig), making linalool the compound of choice for multi-day applications. In summary, linalool ties for best on 3/6 criteria and scores best for 2/6. Thus, based on these six criteria, linalool’s overall performance is superior to currently used anesthetics.

## Discussion

Our results show that linalool is a fast-acting, reversible anesthetic for *Hydra*. It is non-toxic and simple to use, and its pleasant smell makes working with it an enjoyable experience. Incubation in 1 mM linalool does not completely immobilize the animal, as mouth opening and feeding are still observed (Figs 1F and 4A); although, feeding is extremely impaired (Fig 1F, S2 Fig). While *Artemia* frequently get stuck to the tentacles of animals incubated in linalool, few are ingested. This suggests that while nematocyte and mouth function may be normal in linalool-treated animals (Fig 4A), the movement of food into the body cavity is impaired. As digestive movements have previously been shown to require nervous system function [67], this impairment may be a direct consequence of linalool’s effect on the *Hydra* nervous system, as suggested by our data. Our experiments in wildtype and nerve-free animals show the absence of both spontaneous and mechanically induced body column contractions, implying that linalool affects both nervous system and epithelial cells.

In terms of applications, a 10 min incubation in 1 mM linalool significantly decreased polyp movement, allowing for fine surgical manipulations with superior precision, efficiency, and long-term success compared to their execution in HM (Fig 2). Thus, linalool is a useful tool for grafting and tissue manipulations, especially for novice researchers. We achieved significantly improved fluorescent imaging when compared to HM and were able to acquire high quality single- and multi-channel fluorescent z-stacks and time lapse movies (Fig 4 and S3–S6 Movies). Comparable high-quality fluorescence *in vivo* imaging has previously only been possible using chloretone [42], which, as we show here, is not without negative side effects (Fig 8 and S9 Fig), or using custom microfluidics [41] and Polydimethylsiloxane (PDMS) chips [68].

Additionally, we showed that linalool enables repeated short-term imaging of the same specimen over the course of days, allowing us to visualize the dynamics of graft development and head regeneration in individuals (Fig 2Diii and Fig 5A). We were able to achieve fluorescent imaging with sub-cellular resolution (Fig 3), which suggests that one could study cellular migration processes over the course of days. Furthermore, induced mouth opening and induced local body column contraction in linalool treated epithelial GCaMP animals demonstrated that linalool does not interfere with epithelial calcium signaling and that GCaMP animals can be used to study behaviors that are not suppressed by linalool. Therefore, given linalool’s lack of toxicity and ease of use, linalool is likely to become a popular tool by making *in vivo* fluorescence imaging over broad contexts accessible to *Hydra* researchers.

When compared to other currently used anesthetics, 1 mM linalool is superior in terms of ease of preparation, handling, and disposal. Linalool and heptanol are alcohols and supplied as liquids; thus, working solutions are made up within minutes. Because heptanol has a strong smell, however, preparation in the fume hood may be preferred. Urethane and chloretone are powders and therefore the preparation of stock solutions requires more time and safety precautions, such as working in a fume hood. Chloretone also needs to be heated to dissolve. Regarding overall toxicity, linalool is considered non-toxic at the concentrations employed here [69]. Chloretone is also comparably non-toxic at the concentrations used here [70], whereas urethane is a known carcinogen [71–73]. Heptanol is a teratogen [74] and considered to have aquatic toxicity [75], and long-term low-dose exposure causes abnormal patterning phenotypes, such as two-headed animals in freshwater planarians [76]. Thus, linalool provides a clear advantage in terms of ease of use and lack of toxicity.

Linalool also has an advantage in anesthetized animal morphology (Fig 7A). Animals extend in linalool and in urethane and maintain their shape for at least 1 hour, facilitating precision cuts and grafting experiments (Fig 2); in contrast, animals in heptanol or chloretone appear contracted and misshapen and change morphology over time (Fig 7Aii and 7Aiv). As grafting requires precise manipulations that are most easily executed on an evenly extended animal, chloretone and heptanol are suboptimal for such applications. The morphological differences that we observed between the various anesthetics can be important when choosing an anesthetic for a specific application. The demonstrated lack of cellular damage or other harm to the animal with linalool provides an advantage for repeated imaging or for particularly sensitive experiments.

In terms of long-term applications, we find that a 3-day continuous exposure without media exchange is lethal in urethane within 24 h, partially lethal in chloretone, and harmless in linalool and heptanol. Surviving chloretone and heptanol-treated animals showed normal morphology and pinch response, whereas linalool-treated animals do not. The observed detrimental effect on animal health of urethane may be due to an overly broad mechanism of action that impacts other aspects of the animal’s physiology. Urethane has been shown to act in *Hydra* by reversing the sodium polarity across the cell membrane, leading to structural damage [34]. While chloretone has been proposed to act directly on nerves [44], its mechanism of action in *Hydra* remains unclear. It is possible that the gross anatomical changes that are observed in exposure to chloretone cause functional problems that ultimately cause death. Heptanol is a gap junction blocker that effectively blocks ectodermal epithelial cell-cell communication in the body column at 0.04% v/v [45]. As a small alcohol, its effect may be lost over long incubations due to its volatility. Exchanging the media every 24 h drastically changed the outcome of incubation in chloretone and heptanol, with all chloretone-treated and most heptanol-treated animals dying within 3 days. This result suggests that the survival and loss of anesthesia seen in animals incubated 72 h in heptanol or chloretone without medium changes is due to evaporation or degradation of the chemical, and that continuous exposure to active concentrations is toxic to the animals. In summary, these data suggest that urethane, chloretone, and heptanol cannot be used for continuous 3-day exposure and long-term imaging. Thus, for long-term experiments, linalool is the only viable option among the four anesthetics tested.

In contrast to intact polyps, regenerating body columns continuously exposed to 1 mM linalool over 3 days showed abnormal morphology (Fig 6Eii) and suppressed regeneration (Fig 6D). Both, head and foot regeneration were delayed (Fig 6E and S7 Fig). Affected animals healed their wounds but did not develop the structures associated with the missing body part–decapitated animals mostly did not form tentacles or hypostomes, and animals lacking feet did not regain a peduncle or the ability to adhere to the substrate. A small fraction of animals showed one or two tentacle buds at 3 days of regeneration in 1mM linalool (S6 Fig), whereas some animals died under the same conditions, suggesting sensitivity differences among individuals of the same strain. Regeneration could be rescued by transferring the regenerating animals back to HM after linalool exposure (Fig 6E). While these findings prevent the use of linalool for continuous long-term imaging of regeneration, they indicate that linalool could potentially be a useful tool for regeneration studies if the mechanism of action can be elucidated.

Because nerve-free animals in linalool do not show delayed regeneration (S8 Fig), these data suggest that nerve or interstitial cells are the target for the regeneration defect. The precise role of the interstitial cell lineage in regeneration and morphogenesis is unknown. Nerve-free *Hydra* are capable of regeneration and budding [77]. Marcum and Campbell propose several possible explanations for this observation– 1. that nerve cells are not involved in development, 2. that nerve cells modulate developmental processes initiated by epithelial cells, 3. that nerve cells play an essential role in patterning but that their absence can be compensated for, or 4. that nerve and epithelial cells both have critical but overlapping roles in development. Head regeneration is delayed in *Hydra* treated with double-stranded RNA from a gene encoding a neuronal progenitor marker [62]. The authors take this result to support the third possibility laid out by Marcum and Campbell–that neurons are critical for regeneration, but that in their complete absence nerve-free animals can employ an alternate pathway. Our finding that linalool strongly suppresses head regeneration in wild type animals while having no effect on nerve-free animals supports this idea that neuronal signals play an important role for head regeneration under normal circumstances. It will be exciting to dissect this relationship between nerve signaling and axial patterning. One possible starting point for investigation is linalool’s known mechanism of action in other systems.

Linalool has been found to inhibit glutamatergic signaling in the central nervous system [78] and to modify nicotinic receptors at neuromuscular junctions in rodents, leading to modulated acetylcholine release [79]. While it is unclear whether the mechanism of anesthesia in *Hydra* is the same as that in rodents, the cellular machinery targeted is sufficiently conserved that this is a possibility. *Hydra* has been shown to possess GABA receptors [80], and to have specific glutamate-binding abilities likely corresponding to at least two types of glutamate receptors [81]. GABA, glutamate, and their agonists and antagonists have been shown to influence behaviors such as contraction bursts [82], as well as nematocyst activity [83]. On the other hand, *Hydra* homogenate was found to contain an enzyme that hydrolyzes acetylcholine [84]. Nicotinic acetylcholinesterase antagonists were found to decrease contraction bursts while a muscarinic acetylcholinergic antagonist increased them [85]. A cDNA sequence for acetylcholinesterase has also been cloned, though its expression and localization have not been confirmed [37]. Thus, the targets of linalool’s mechanism of action appear to be conserved between *Hydra* and rodents, though further mechanistic studies will be needed to confirm a shared mode of action.

Finally, because different species are used in *Hydra* research, we also tested the suitability of linalool as a reversible anesthetic in other *Hydra* species–*H*. *oligactis* and *H*. *viridissima*. The results for induction and recovery times and effects on pinch and feeding responses are summarized in S10 Fig. We found similar effects as observed for *Hydra vulgaris* (S10 Fig) and conclude that linalool effectively anesthetizes these other *Hydra* species.

In summary, linalool offers a range of advantages over other available anesthetics by enabling new applications such as long term or repeated imaging while also being usable as a pre-fixation relaxant in the same way as current options. Linalool’s lack of toxicity to both *Hydra* and researchers and the ease of use and preparation compared to current anesthetics render it an attractive tool for *Hydra* experimentation in the teaching setting. Excitingly, linalool makes grafting experiments that can provide fundamental insights into regeneration and biological patterning accessible to students with no previous experience with *Hydra*.

## Materials and methods

### *Hydra* strains and culture

We used the *Hydra vulgaris* AEP strain [86,87] and various transgenic lines derived from this strain: GCaMP6s, expressing the calcium sensor GCaMP6s in interstitial cells [16]; Epithelial GCaMP, expressing GCaMP6s in the endoderm cells [58], Wnt, expressing GFP under control of the Wnt3 promoter [88]; HyBra, expressing GFP under control of the HyBra2 promoter [89]; “Watermelon” (WM) animals [89] expressing GFP in the ectoderm and DsRed2 in the endoderm with both genes under control of an actin gene promoter; *Hydra vulgaris* strain A10 (chimera consisting of *Hydra vulgaris* (formerly *Hydra magnipapillata* strain 105) epithelial cells and sf-1 interstitial cells, which are temperature sensitive interstitial cells [90]; and a line originating from a single animal that was obtained by recombining AEP ectoderm and watermelon endoderm following tissue separation [11] and named “Frank” by the undergraduate student who created it. The Frank line has unlabeled ectoderm and DsRed2-expressing endoderm. *Hydra viridissima* and *Hydra oligactis* were generously provided by Dr. Rob Steele.

*Hydra* strains were maintained in mass cultures in *Hydra* medium (HM) composed of 1 mM CaCl_2_ (Spectrum Chemical, New Brunswick, NJ), 0.1 mM MgCl_2_ (Sigma-Aldrich, St. Louis, MO), 0.03 mM KNO_3_ (Fisher Scientific, Waltham, MA), 0.5 mM NaHCO_3_ (Fisher Scientific), and 0.08 mM MgSO_4_ (Fisher Scientific) prepared with MilliQ water, with a pH between 7 and 7.3. Cultures were maintained at 18°C in the dark in a Panasonic incubator (Panasonic MIR-554, Tokyo, Japan) save the *H*. *oligactis* and *H*. *viridissima* which were kept on a windowsill at room temperature. The cultures were fed 2-3x/week with *Artemia* nauplii (brine shrimp) from the San Francisco Bay or from the Great Salt Lake (Brine Shrimp Direct, Ogden, UT). Animals were cleaned daily using standard cleaning procedures [91]. Asexual, non-budding polyps starved for at least 24 h were used for experiments unless stated otherwise.

### Generation of nerve-free *Hydra*

To generate nerve-free *Hydra*, A10 polyps were heat-shocked in an incubator (Fisher Scientific 615F) at 28–29°C in the dark for 72h and then moved back into the 18°C incubator [67,92,93]. All nerve-free animals were subsequently force-fed and “burped” as described previously [94] for three to four weeks, in which time they lost nematocytes, as well as feeding and mouth opening behaviors.

### Preparation of anesthetic solutions

Stock solutions were made in HM at concentrations of 1 mM linalool (Sigma-Aldrich), 0.04% heptanol (Acros Organics, Fisher Scientific), 2% urethane (Sigma-Aldrich), or 0.1% chloretone hemihydrate (Sigma-Aldrich). Linalool and heptanol were prepared fresh daily by adding to HM and shaking vigorously for 1 min to dissolve and, stored at room temperature. Urethane and chloretone solutions were stored at 4°C for a few days and pre-warmed to room temperature before usage. Anesthetic solutions were prepared at room temperature, except for chloretone, which was prepared with slight heating.

### Linalool viability assay

24 h starved polyps were incubated in 6-well plates (Genesee Scientific, El Cajon, CA), 8 or 10 animals per well in 2 mL of different concentrations of linalool (0–10 mM) at room temperature for 3 h. Below concentrations of 3 mM and above 3.6 mM, 4 technical replicates were performed for each concentration. Between 3 mM and 3.6 mM, 6 technical replicates were performed at each concentration. The fraction of live animals was scored at the end of the assay. To obtain the LC50 value, the fraction of dead animals (1- fraction of live animals) was plotted against the linalool concentration. The data were fitted to the Hill equation as in [53]:

$$y=\frac{1}{1+{\left(\frac{LC_{50}}{x}\right)}^{Hill-coefficient}}$$

Here, y is the fraction of dead animals and x is the concentration of linalool in millimolar. The fit was generated using the curve fitting application in MATLAB (MathWorks, Natick, MA, USA) to obtain the mean LC50 value and the 95% confidence intervals.

### Characterizing short term efficacy of anesthetics

1–5 intact *Hydra* polyps were incubated per well in a flat bottom 6-well plate (Eppendorf, Hamburg, Germany) filled with 8mL of HM or respective anesthesia. If more than 2 polyps were used, 40 μm or 100 μm Falcon cell strainers (Fisher Scientific) were used in the well to allow for quicker transfer of the animals from HM to anesthesia and vice versa. In some experiments, all wells were imaged simultaneously, and polyps were stained with neutral red (1:400,000 w/v; Fisher Scientific) in HM for 90 s at room temperature prior to the experiment to enhance contrast during imaging. The 6-well plate was imaged from the top using a Basler A601f-2 camera (Basler Inc., Exton, PA) attached to a 25 mm TV lens C22525KP with adjustable focal length (Pentax, Tokyo, Japan) for 65 min at 1 fps using Basler pylon camera software. Lighting was provided by a model A4S light box (ME456, Amazon, Seattle, WA). After 65 min, the cell strainers were moved to a new well and 8 mL of HM were added to each well. Following this, the plate was imaged for 2 h. In other experiments, individual wells were imaged on a stereo microscope using a Flea-3 camera (FLIR Integrated Imaging Solutions Inc, Wilsonville, OR) controlled by a custom MATLAB script. To obtain representative images at higher magnification, anesthetized *Hydra* were imaged in a 35 mm tissue culture dish with a Leica MZ16FA microscope (Leica Microsystems Inc., Buffalo Grove, IL) equipped with a SPOT RT3 camera (SPOT Imaging, Sterling Heights, Michigan), using the SPOT 5.1 software (SPOT Imaging) at 15 min and at 60 min exposure.

A range of sublethal linalool concentrations (0 mM, 0.1 mM, 0.25 mM, 0.5 mM, 0.75 mM,1 mM) were tested. Working concentrations for other anesthetics were 2% urethane, 0.04% heptanol, or 0.1% chloretone, with induction imaged for at least 20 min and recovery for at least 30 min. At least 10 animals were assayed for each condition, in at least 3 technical replicates. Time of induction of anesthesia was considered to be the time at which the animal stopped extending further after its last spontaneous contraction, and time of recovery was considered to be the timing of complete contraction in the first contraction burst observed after returning the polyps to HM. Due to the complex behavior of *Hydra* and the subjectivity of these measures, calculated times for induction and recovery should be considered estimates rather than conclusive values.

### Body column length of *Hydra* in anesthetics

24 h starved polyps were imaged for 10 min in HM to observe both extended and contracted states of the moving polyp to calculate an average body length ((max+min)/2). The polyps were then transferred to 1 mM linalool, 2% urethane, 0.04% heptanol, or 0.1% chloretone and imaged for an additional 20 min. We averaged the minimum and maximum body lengths of *Hydra* in the last 10 min of recording in each anesthetic. Average body length in the anesthetic was divided by the average in HM to find the % body length for each anesthetic to determine whether the polyps were hyperextended (>100%) or contracted (<100%) compared to their “normal” length. Because *Hydra* doesn’t have a fixed body shape or length due to constant extension and contraction, this normal length is somewhat arbitrary; it nevertheless allows us to compare the effects of the various anesthetics.

### Feeding and pinch responses in linalool

24 h starved polyps were incubated in 1 mM linalool for 10 min in a 60 mm tissue culture dish (VWR International, Radnor, PA). Each animal was pinched using a pair of Dumont No. 5 forceps (Fine Surgical Tools, Foster City, CA) to determine presence or absence of a contractile response while in the linalool solution.

To evaluate how quickly the pinch response would be restored after removal from 1mM linalool, six 1-day starved *Hydra* were incubated in 1mM linalool for either 10min or 30min in a 30mm diameter dish. After incubation they were moved over to the lid of a 30mm dish containing 3 ml Hydra medium with a glass pipet and their behavior was recorded on a Leica Wild M3C dissection microscope, equipped with a Flea-3 camera. Three independent replicates were performed for each 10 min and 30 min exposure, with 6 biological replicates per technical replicate.

To assay whether animals exhibited a feeding response in linalool, 4-day starved polyps were first incubated for 10 min in HM or 1 mM linalool. Meanwhile, 30 *Artemia* were counted and added to a well of a 96-well plate (Eppendorf) either in HM or 1mM linalool. A picture of the well was taken using a Flea-3 camera attached to a stereo microscope to record the number of *Artemia* in the well. A single polyp was then added to the well and left undisturbed for 30 min. At the end of 30 minutes, the number of *Artemia* ingested were counted, either by pulling the ingested *Artemia* out of the animal’s body cavity using forceps in the case of the *H*. *viridissima* or by counting the number of freely floating *Artemia* in the dish.

To assay how long anesthetized animals took to regain a feeding response once removed from linalool, 5 animals were incubated in 1mM linalool for 10-15min. A single individual was added to a small drop (60–80μl) of HM containing approx. 70–80 *Artemia*. All 5 animals were imaged using a Leica dissection microscope and Flea-3 camera at regular intervals to determine whether the *Hydra* started eating *Artemia*. The number of animals eating at 5, 10, and 15min were recorded. Eating was determined as having an enlarged body column due to ingestion of *Artemia*. Three technical replicates with 5 animals each were performed.

### Cross sections and “zebra grafts”

48–72 h starved Wnt and Frank polyps were used to assay sectioning. Polyps were placed in the lids of 35 mm dishes in either HM or 1 mM linalool for at least 10 min. Rings of tissue were excised from the body column using a scalpel 10 blade. The rings were strung onto glass needles pulled from microcapillaries (World Precision Instruments, Sarasota, FL) using a P-1000 micropipette puller (Sutter Instrument, Novato, CA) and imaged with a Leica MZ16FA microscope equipped with a SPOT RT3 camera, using the SPOT 5.1 software. The time taken to cut the sections was measured for each polyp. The thickness of each section was measured using Fiji [95] by measuring the length of the thickest part of the cross section.

“Zebra grafts” (n = 10 per condition) were created using WM and Frank animals. One animal of each kind was used to make one graft. The animals were placed in a 100 mm petri dish (Spectrum Scientifics, Philadelphia, PA) filled with either HM or 1 mM linalool in HM. A small piece of filter paper (2x2 mm) was cut and threaded onto a size 00 enameled insect pin (Austerlitz, Carolina Biological) and the pin placed into the dish. One animal was decapitated, and the head threaded onto the pin mouth first using forceps such that the cut edge of the tissue faced towards the point of the pin. The second animal was then decapitated, and the head discarded. A ring of tissue was cut as thinly as possible from the body column of the second animal and threaded onto the pin, followed by a ring from the first animal. Alternating rings of tissue were cut and placed on the pin until the body columns of both animals were used up, at which point one of the feet was threaded onto the pin to complete the chimera. A second piece of filter paper was threaded onto the pin, and forceps used to gently move the two pieces of paper together in order to force all the rings into contact with each other. These chimeras were allowed to heal on the pins for 2 h, then gently pushed off the pins with forceps, transferred to clean 35 mm dishes full of HM, and allowed to further heal overnight before imaging. Two grafts were then imaged using an Invitrogen EVOS FL Auto microscope (Thermo Fisher Scientific) and the Invitrogen EVOS FL Auto Imaging System software. Since the entire graft did not fit in a single field of view, multiple images were taken and stitched together in Inkscape 0.92.3 which is an opensource vector graphics editor. The other grafts were imaged using an Olympus IX81 inverted microscope (Olympus Corporation, Tokyo, Japan) with an ORCA-ER camera (Hamamatsu Photonics, Hamamatsu, Japan) and slidebook software version 5.0 (Intelligent Imaging Innovations, Denver, CO). The number of segments in each graft and the time taken to cut sections of the parent animals and assemble the graft on the pin was recorded.

Grafting of heads into the body column was accomplished using WM and unlabeled animals using an approach similar to the insect pin method described above. The WM animal was decapitated, and a slit cut in the side of the unlabeled animal. The pin was passed through the WM head hypostome first, then through the wound in the unlabeled polyp and out through the body wall on the other side. Care was taken when positioning the filter paper pieces to avoid pushing the donor head into the recipient body cavity. Animals were allowed to heal for 2 h, then removed from the pins and placed in dishes of clean HM to heal overnight before imaging. Grafting of head organizers into the body column was accomplished without pins. Head organizers were obtained by anesthetizing a WM animal in linalool, removing the head, then excising the tentacle bases to leave only a small fragment of tissue containing the tip of the hypostome. A small slit was cut in the body column of an unlabeled animal, and forceps used to place the hypostome piece into the slit. Animals were allowed to heal for 2 h before transfer to dishes of clean HM. Successful grafts were imaged every 24 h to determine whether an ectopic body axis was induced.

### Fluorescence imaging in 1 mM linalool and in other anesthetics

All fluorescence imaging was done using the Olympus IX81 inverted microscope with the ORCA-ER camera. Slidebook software was used to interface with the microscope and acquire z-stacks and time-lapse images. Anesthesia incubations were performed as described earlier.

*Hydra* expressing GCaMP6s in nerve cells and WM *Hydra* were used for fluorescence imaging. For low magnification single channel imaging, a GCaMP6s animal was allowed to move freely in a drop of either HM or 1 mM linalool on a 40 mm x 24 mm glass coverslip (Fisher Scientific) and was imaged in the GFP channel with a 50 ms exposure using a 4x objective (Olympus). Images were recorded every 100 ms for 10 s to obtain a time lapse movie. Rigid body correction of z-stacks was accomplished using a previously described algorithm [96]. For high-magnification single channel imaging, GCaMP6s animals were mounted in tunnel slides prepared as described in [32]. Neurons in the body column of GCaMP6s animals were imaged by taking z-stacks of the tissue in the GFP channel (500 ms exposure; z-step size of 0.25 μm), using a 60x oil immersion objective (Olympus).

For imaging calcium activity during mouth opening, 4–5 day starved epithelial GCaMP animals were incubated in 1 mM linalool for 10 minutes. The animals were then decapitated and the hypostomes mounted in tunnel slides. Mouth opening was induced by flushing in 2 mM reduced glutathione (Sigma-Aldrich) diluted in 1 mM linalool into the tunnel slide [32]. Images were taken every 400 ms with an 80 ms exposure using a 10x objective (Olympus) in the GFP channel.

For calcium imaging using a pinch response, 5 epithelial GCaMP animals were used. A single polyp was moved into a 60 mm petri dish with HM. Response to pinch was recorded on the Leica MZ16FA microscope equipped with the SPOT RT3 camera at 5 fps. The same polyp was then moved into a second 60 mm petri dish containing 1 mM linalool and incubated at room temperature for 10 minutes. Pinch response was then recorded in the same way it was done for HM.

For low-magnification multi-channel imaging, WM animals were incubated in Hoechst 33342 (Thermo-Fisher Scientific) diluted 1:500 in 1 mM linalool for 15 minutes in the dark. The animals were then decapitated and the hypostome mounted in a tunnel slide. Z-stacks were taken in DAPI, GFP and RFP channels with a step size of 2.99 μm using a 10x objective.

For high-magnification multi-channel imaging, RWM animals were first incubated in SYTO 60 red fluorescent nucleic acid stain (Invitrogen) diluted to 10 μM in HM for 1 h at room temperature in the dark. 2 quick washes in 1 mL HM followed, as well as a15 min incubation in the dark at room temperature in 1:250 Hoechst 33342 diluted in 1 mM linalool. Body columns of the animals were imaged in the DAPI, RFP and DRAQ5 channels with a 60x oil immersion objective. For high magnification two-channel imaging, Hoechst 33342 (Thermo-Fisher Scientific) was diluted 1:500 in 1 mL of the respective anesthetic solution and WM animals were incubated for 15 min at room temperature in the dark. Individuals were mounted on tunnel slides and imaged.

For long term imaging, whole polyps were placed in 35 mm plastic dishes in 2 mL 1 mM linalool solution. They were imaged unconstrained with no stage movement once every 5 minutes for 24 h using an Invitrogen EVOS FL Auto microscope.

### Quantification of movement of samples during imaging

To quantify the amount of movement of the samples during imaging, the z-stacks obtained by imaging GCaMP6s animals at 60x and t-stacks obtained by imaging them at 4x were projected using a SD projection in Fiji. The mean gray value of the projection was obtained and divided by the mean grey value of the first image of the z-stack or t-stack to calculate the coefficient of variation.

### Regeneration and budding assays

Polyps were decapitated with a scalpel just below the tentacle ring for head regeneration and above the budding zone for foot regeneration assays. In one experiment, the decapitated animals were placed in 600 μL of 0 mM (control), 0.1 mM, 0.25 mM, 0.5 mM, 0.75 mM, or 1 mM linalool in HM. Head regeneration was scored by the appearance of the first tentacle on a decapitated animal. 8 animals were kept at each concentration in a 48-well plate (Eppendorf) and imaged in brightfield at 4x with the Invitrogen EVOS Fl Auto 2. Head regeneration was scored every 12 h for 72 h. The lid of the plate was removed for imaging and the solutions were changed every 24 h. In another experiment, decapitated polyps were placed individually into the wells of a 24-well plate (Eppendorf), filled either with 500 μl HM or 1 mM linalool. Polyps were imaged approximately every 12 h and the appearance of tentacles and hypostomes were scored. After approximately 3 days, polyps were transferred into a new 24-well plate containing 500 μl fresh HM and imaged a day after transfer. Foot regeneration experiments were conducted the same way, with animals scored for the appearance of a peduncle and for the ability to adhere to the substrate. For repeated imaging of head regeneration at high magnification, animals were anesthetized in 1 mM linalool for 10 min prior to imaging and returned to HM to recover afterwards. To facilitate removal from the slides, a layer of Scotch tape was placed over the double-sided tape during construction of tunnel slides. The increased space between coverslip and slide and ability to easily lift off the coverslip after imaging allowed recovery of the animal with minimal chance of injury.

Budding was assessed by selecting healthy animals with early buds at stages 3–4 on the previously described scale [97], and incubating them in well plates as described for regeneration assays. Animals were scored for development of tentacles on the bud and formation of further buds at the end of 3 days. Long-term imaging of budding was carried out in 35 mm glass bottomed dishes (MatTek, Ashland, MA). One animal was placed onto the glass surface at the bottom of the dish in 1 mM linalool, a coverslip was laid over the top to constrain the animal, and the dish was flooded with 1 mM linalool. Animals were imaged once per hour for 48 h using an Invitrogen EVOS FL Auto microscope.

### Cell viability assay

Polyps were incubated for 30 min in 1 μg/mL propidium iodide in HM, washed twice in HM, then mounted on glass slides as described for live imaging of neurons. Slides were imaged on an Invitrogen EVOS FL Auto microscope in the red fluorescence channel using the Invitrogen EVOS FL Auto Imaging System software. Labeled cells were counted in the body column only and reported as number of labeled cells per animal. As a positive control, polyps were incubated in 0.04% colchicine (Acros Organics) in HM to induce cell death [98]. Animals were incubated in colchicine for a full 24 h rather than 8 h incubation followed by 16 h recovery as described.

### Mitotic index assay

Polyps were incubated in HM or 1 mM linalool for 72 hours in 60 mm cell culture dishes at a density of 1 polyp/mL. Polyps were not fed during the experiment, but the medium was changed daily. At the end of the 72 h, one or two cross sectional segments were cut from the body column of each polyp near the head. The samples were placed on glass slides for a wet mount antibody stain. Humid chambers for staining were constructed by lining covered 100 mm Petri dishes (Spectrum Scientific) with wet paper towels and placing the slides inside the dishes. A well was created in the center of each glass slide by layering two pieces of double-sided tape across both short sides of the slide with one piece of tape running on both long edges of the slide. The samples were placed in a drop of medium on the slide. All steps were performed at room temperature unless otherwise noted. The samples were fixed in 20 μL 4% paraformaldehyde (Sigma-Aldrich) in HM for 15 min. The samples were washed three times with 20 μL 1x PBS, followed by a 15 min permeabilization with 20 μL 0.5% PBSTx (0.5% Triton-X in 1x PBS). They were then incubated for 3.5 h in 20 μL blocking solution (1% FBS, 0.1% DMSO in 1x PBS) and placed overnight (16h) at 4°C in 30 μL anti-phospho-histone H3 (Ser10) primary antibody (Millipore Sigma, Burlington, MA) diluted 1:100 in blocking solution. On the second day, samples were washed quickly 3x with 40 μL 1x PBS, followed by four 25–35 minute washes of 20 μL 0.3% PBSTx. The samples were then incubated in a 1:1000 or 1:500 dilution of Alexa 546 rabbit IgG secondary antibody (Thermo-Fisher Scientific) for 5 h, followed by three quick and two 10 min washes of 0.3% PBSTx. To stain nuclei, the samples were incubated in DRAQ5 (Thermo-Fisher Scientific) diluted to 5 μM in 1x PBS for 15 min and then washed three times with 1x PBS. The 1x PBS was replaced with a 1:1 solution of glycerol and HM. Finally, a cover slip was placed over the samples and nail polish was used to seal the slides. Z-stacks of the cross-sections were imaged using a Leica high-resonance scanning SP5 confocal microscope with a 20x C-Apochromat 1.2 W objective.

To calculate mitotic indices, the number of Alexa 546 stained nuclei was counted for each cross section, divided by the number of nuclei stained by DRAQ5 and multiplied by 100 to obtain a percentage. Counting of Alexa 546 and DRAQ5 stained nuclei was done using Fiji. For the z-stack corresponding to each color channel, a maximum intensity z-projection was taken and binarized. The projection was then segmented using the water-shedding tool. The number of particles was counted using the *Analyze Particles* tool, with a size range of 10- infinity μm^2^. For Alexa 546 color channel stacks, an additional thresholding step was used before binarizing the image.

### Comparison of different anesthetics

The different anesthetics were ranked 1–4, based on our direct comparison of their performance and the criteria described in Table 2. 1 was considered excellent, 2 good, 3 fair and 4 poor. The anesthetics were ranked the same if the difference in relevant values for comparison were not statistically significant.

**Table 2:** Criteria for ranking anesthetics.

Readout	Criterion
Induction time	Ranked in increasing order of median induction times (Fig 8B).
Recovery time	Ranked in increasing order of median recovery times (Fig 8C).
Lethality	Ranked in decreasing order of fraction of surviving animals at the end of 3d incubations (S9B Fig).
Ease of use	Ranked based on availability as solid or liquid, with liquids ranking higher, and on toxicity (requiring handling in the fume hood or not).
Morphology	Ranked on basis of closeness to appearance of animal in HM. Effects such as bloating, lumps, or stubby tentacles were ranked lower (Fig 7A).
Immobilization	Quality of z-projections of z-stacks of neuronal GCaMP6s animals taken at 60x (Fig 7B).

## Supporting information

S1 Fig Abnormal morphology of *Hydra* after 3 h incubation in linalool. Animals are contracted with stubby tentacles in concentrations of 2 mM and 2.5 mM. Images representative of 5/5 animals imaged at the different concentrations. HM denotes *Hydra* medium control. Scale bar: 1mm.
(TIF)

S2 Fig *Hydra* pinch and feeding responses after recovery from 1 mM linalool. A. Animals display a normal pinch response after 5 min recovery in *Hydra* medium (HM). Image representative of 18/18 polyps across 3 technical replicates. Scale bar: 0.5 mm. B. Feeding after 0, 5, 10, and 15 min recovery in HM. Scale bar: 1mm. C. Percentage of animals that feed after 5 min (47 ± 31% (mean ± SD)), 10 min (60 ± 20%) and 15 min (73 ± 12%) recovery in HM (averages over 3 technical replicates with 5 polyps each). Error bars represent SD. D. Median number of shrimp ingested by each animal incubated in HM for 30 minutes was 13 (11, 16; 25^th^ percentile, 75^th^ percentile) for n = 9 polyps across 2 technical replicates. On the other hand, only 2 out of 9 animals (across 2 technical replicates) kept in 1 mM linalool ingested shrimp in the 30 min. Both animals ingested only one shrimp each. (***) denotes statistically significant difference at p < 0.001 (Mann- Whitney U test).
(TIF)

S3 Fig Linalool improves outcome of zebra grafts. A. Zebra grafts conducted in *Hydra* medium (HM), imaged 24 h after grafting. Scale bar: 1 mm. B. Zebra grafts conducted in 1 mM linalool, imaged 24 h after grafting. Scale bar: 1 mm.
(TIF)

S4 Fig Hypostome graft morphologies. Animals that retained the grafted tissue or formed an ectopic axis, with grafting performed in either *Hydra* medium (HM) or 1 mM linalool (from n = 17 attempts per condition) are shown. Scale bars: 0.5 mm.
(TIF)

S5 Fig Linalool does not impact budding rate. A. Bud development in budding animals incubated continuously for 3d in HM or 1 mM linalool, 30 animals per condition across 3 technical replicates. There was no statistically significant difference between animals in HM and in linalool (2-tailed t-test). Error bars represent SD. B. Representative image of animal with fully developed bud. C. Representative image of animal with two buds. Scale bar: 1 mm.
(TIF)

S6 Fig Head regeneration in linalool. 14/42 decapitated polyps across 4 technical replicates regenerated small tentacle buds at the end of 3d incubation in linalool. Linalool solution was changed every day. The remaining animals did not regenerate head structures. Scale bar: 0.5 mm.
(TIF)

S7 Fig Linalool inhibits foot regeneration. A. i. Three-day incubation in linalool prevents foot regeneration. Data from 36 polyps per condition across 3 technical replicates. (***) denotes statistically significant difference between percentage of animals with regenerated foot in HM and linalool at p < 0.001 (Fisher’s exact test) when comparing overall numbers. ii. Phenotype is rescued after 3d recovery in HM. Data from 30 polyps per condition across 3 technical replicates. B. Polyp incubated 3d in HM after foot amputation. C. Polyp incubated 3d in 1 mM linalool after foot amputation. Scale bar: 1 mm.
(TIF)

S8 Fig Head regeneration in nerve-free *Hydra* is not negatively impacted by 5-day incubation in linalool. A. Representative images of nerve-free polyps regenerating their heads in i. HM and ii. linalool after 4d incubation. Scale bar: 1 mm. B. Percentage of animals with at least one regenerated tentacle over time (n = 10 animals in 2 technical replicates). There is no statistically significant difference between animals regenerating in HM compared to those regenerating in linalool (Fisher’s Exact test).
(TIF)

S9 Fig Lethality of 3d incubation in various anesthetics. A. Incubation without changing media. n = 20 animals per condition across 3 technical replicates. Surviving heptanol and chloretone animals had a normal pinch response at 3d. Surviving linalool animals remained anesthetized. B. Incubation with media exchanged every 24h. n = 22 animals per condition across 3 technical replicates except for linalool and urethane where 2 technical replicates with 5 animals per replicate were performed. C. Statistical comparison of number of surviving animals at each time point in each anesthetic (without media changes) with the HM control as reference (Fisher’s Exact test). D. Statistical comparison of number of surviving animals at each time point in each anesthetic (with media changes) with the HM control as reference (Fisher’s Exact test). E. Pairwise statistical comparisons of number of animals surviving at the end of the 3d incubation in the anesthetics (with media changes) (Fisher’s Exact test). (C-E) Pink, red and dark red indicate a statistically significant difference at p<0.05, p<0.01 and p<0.001 respectively, determined using the Fisher’s Exact test between pairs of anesthetics.
(TIF)

S10 Fig Response of *H*. *oligactis* and *H*. *viridissima* to 1 mM linalool. A. Time for induction of anesthesia, measured as time of full extension after last observed contraction burst, in 1 mM linalool (n = 9 for *H*. *oligactis* across 3 technical replicates, n = 12 for *H*. *viridissima* across 3 technical replicates). B. Time for recovery from anesthesia, measured as time of first observed contraction burst, after being moved to HM from 1 mM linalool (n = 11 for *H*. *oligactis* across 3 technical replicates, n = 12 for *H*. *viridissima* across 3 technical replicates). C. Feeding assay. Feeding is inhibited due to linalool incubation. While animals in linalool have shrimp stuck to their tentacles, they do not have any in their body column, contrary to what is seen with animals in HM. Scale bar 0.5 mm. D. Pinch responses in HM and 1 mM linalool. Pinch response is inhibited by linalool incubation. Scale bar 1mm.
(TIF)

S1 Movie 1 mM linalool prevents pinch response. Time-lapse movie of *Hydra* polyp incubated in either *Hydra* medium (HM; left) or in 1 mM linalool for 10 minutes (right). Experimental details provided in Methods in main text. Movie is representative of 10/10 animals across 2 technical replicates. Video playback: 1fps. Scale bar: 1mm.
(AVI)

S2 Movie 1 mM linalool prevents pinch response in nerve-free animals. Time-lapse movie of nerve free *Hydra* polyp incubated in either *Hydra* medium (HM; left) or in 1 mM linalool for 10 minutes (right). Experimental details provided in Methods in main text. Movie is representative of 6/6 tested nerve-free animals across 2 technical replicates. Video playback: 1fps. Scale bar: 1mm.
(AVI)

S3 Movie Linalool incubation allows for low magnification time-lapse imaging. Time-lapse movie of freely moving GCaMP6s animals in either *Hydra* medium (HM; left) or incubated for at least 10 min in 1 mM linalool (LL; right). Experimental details are provided in Methods in the main text and Fig 3A shows a Maximum Intensity Projection of the video. Video playback: 10fps. Scale bar: 100 μm.
(AVI)

S4 Movie Linalool allows for high magnification time-lapse imaging. Time-lapse movie showing a z-stack through the body column of a GCaMP6s animal in either *Hydra* medium (HM; left) or incubated for at least 10 min in 1 mM linalool (LL; right). Experimental details are provided in Methods in the main text and Fig 3B shows a single slice and the Maximum Intensity Projection of the video. Video playback: 10fps. Scale bar: 10 μm.
(MP4)

S5 Movie Linalool treatment allows for multi-channel fluorescence time-lapse imaging. Time-lapse movie of a 3-channel acquisition of a watermelon animal stained with Hoechst nuclear dye. The head was mounted as described in Carter *et al*. [32] in 1 mM linalool and flushed with 2 mM reduced glutathione to trigger a feeding reaction. While the mouth stays closed during recording, one can clearly see the quality of imaging that can be obtained in linalool, allowing for simultaneous imaging of cell shapes and nuclear positions. Video playback: 10fps. Scale bar: 100 μm.
(AVI)

S6 Movie Linalool does not interfere with calcium imaging. Time-lapse movie showing calcium activity in epithelial GCaMP animal during chemically induced mouth opening. The head was mounted as described in Carter *et al*. [32] in 1 mM linalool and flushed with 2 mM reduced glutathione to trigger a feeding reaction. Video playback: 10fps. Scale bar: 100 μm.
(MP4)

S7 Movie Linalool does not sufficiently immobilize polyps for long term imaging. Shown first, an unconstrained animal in *Hydra* medium, which exits the field of view within 90 min of recording. In contrast, it is possible to take a 24 h time lapse movie of an unconstrained *Hydra* in 1 mM linalool. However, while linalool incubation significantly improves stability for imaging, the animal moves too much for experiments requiring cellular resolution. Video playback: 10fps. Scale bar: 0.5 mm.
(MP4)

S8 Movie Calcium activity in response to pinching. Time-lapse movie showing calcium activity in epithelial GCaMP animal in response to pinching. Animal in *Hydra* medium (HM) shows the global contraction accompanied by calcium activity in the entire body column. Animal incubated in linalool only shows calcium activity at the site of pinching. Video playback: 10fps. Scale bar: 1mm.
(AVI)

S1 Table Coefficient of variation. Calculated for t-stacks of GCaMP animals imaged at 4x and z-stacks of GCaMP animals imaged at 60x, in 1 mM linalool and in HM. (**) indicates statistically significant difference from corresponding imaging in Hydra medium at p < 0.01 as determined by a two-tailed t-test.
(DOCX)
