# Supplementary figures and images for: Linalool acts as a fast and reversible anesthetic in Hydra
Source: PLoS One. 2019 Oct 24;14(10):e0224221. doi: 10.1371/journal.pone.0224221 (PMC6812832; doi:10.1371/journal.pone.0224221)

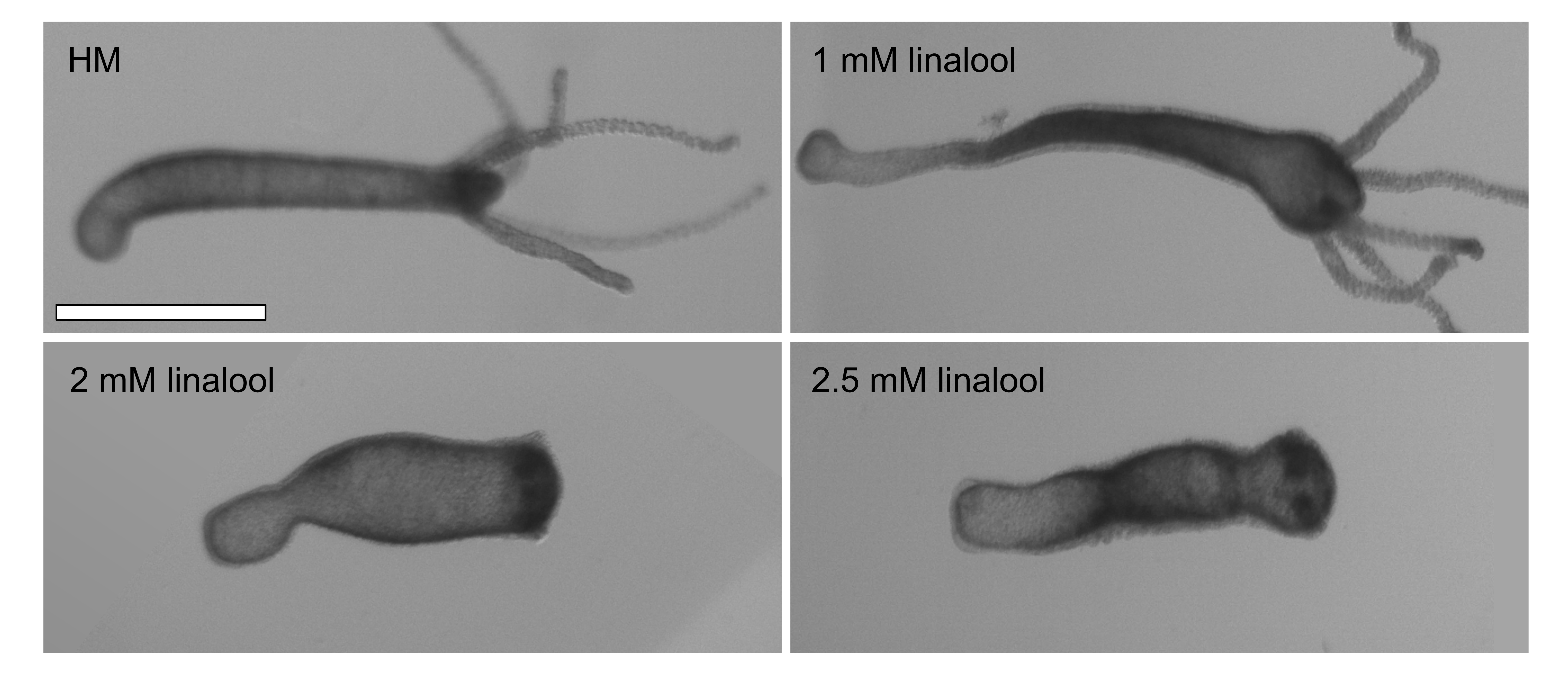

Supplement: S1 Fig — Animals are contracted with stubby tentacles in concentrations of 2 mM and 2.5 mM. Images representative of 5/5 animals imaged at the different concentrations. HM denotes Hydra medium control. Scale bar: 1mm. (TIF) [file pone.0224221.s001.tif]

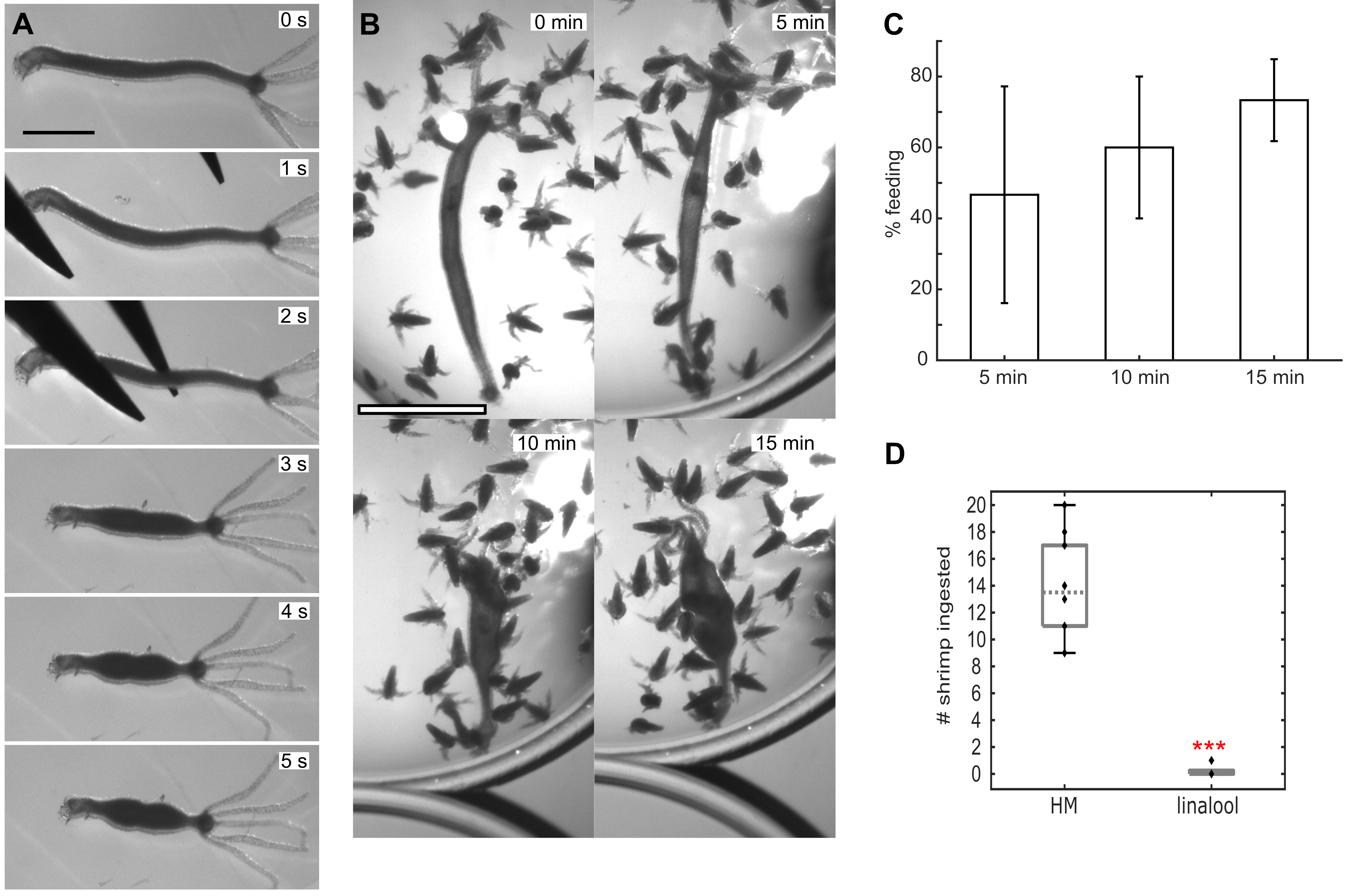

Supplement: S2 Fig — A. Animals display a normal pinch response after 5 min recovery in Hydra medium (HM). Image representative of 18/18 polyps across 3 technical replicates. Scale bar: 0.5 mm. B. Feeding after 0, 5, 10, and 15 min recovery in HM. Scale bar: 1mm. C. Percentage of animals that feed after 5 min (47 ± 31% (mean ± SD)), 10 min (60 ± 20%) and 15 min (73 ± 12%) recovery in HM (averages over 3 technical replicates with 5 polyps each). Error bars represent SD. D. Median number of shrimp ingested by each animal incubated in HM for 30 minutes was 13 (11, 16; 25th percentile, 75th percentile) for n = 9 polyps across 2 technical replicates. On the other hand, only 2 out of 9 animals (across 2 technical replicates) kept in 1 mM linalool ingested shrimp in the 30 min. Both animals ingested only one shrimp each. (***) denotes statistically significant difference at p < 0.001 (Mann- Whitney U test). (TIF) [file pone.0224221.s002.tif]

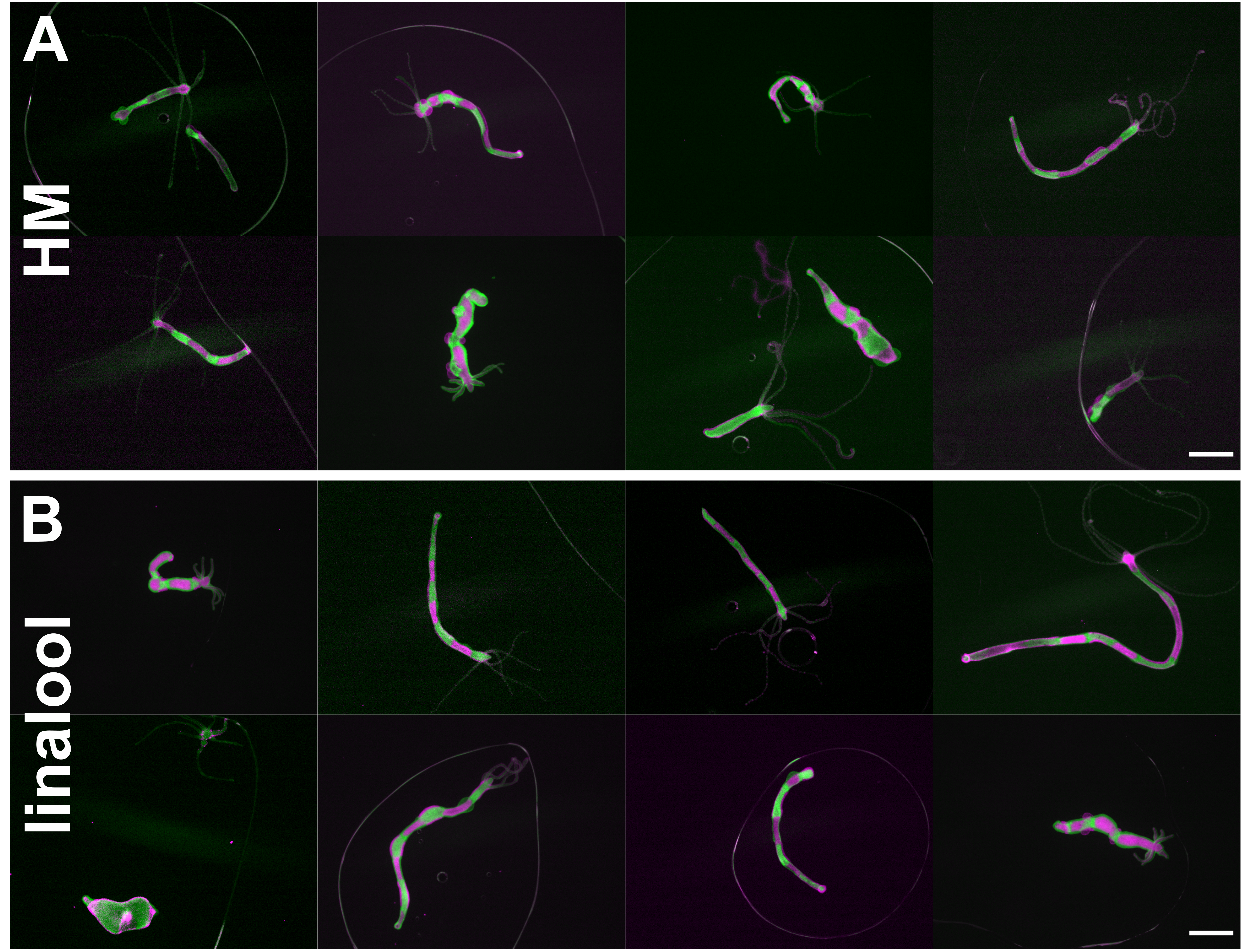

Supplement: S3 Fig — A. Zebra grafts conducted in Hydra medium (HM), imaged 24 h after grafting. Scale bar: 1 mm. B. Zebra grafts conducted in 1 mM linalool, imaged 24 h after grafting. Scale bar: 1 mm. (TIF) [file pone.0224221.s003.tif]

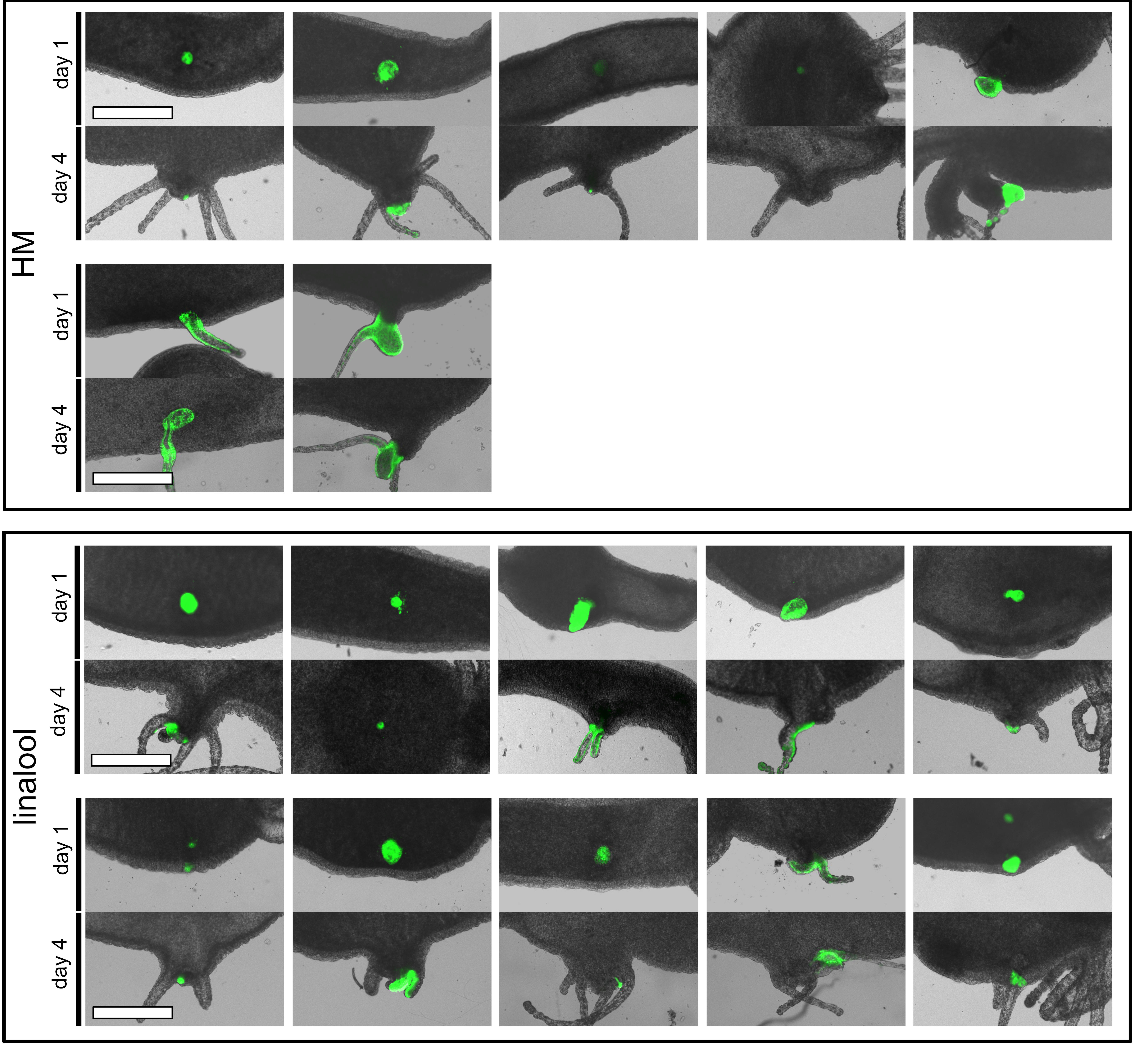

Supplement: S4 Fig — Animals that retained the grafted tissue or formed an ectopic axis, with grafting performed in either Hydra medium (HM) or 1 mM linalool (from n = 17 attempts per condition) are shown. Scale bars: 0.5 mm. (TIF) [file pone.0224221.s004.tif]

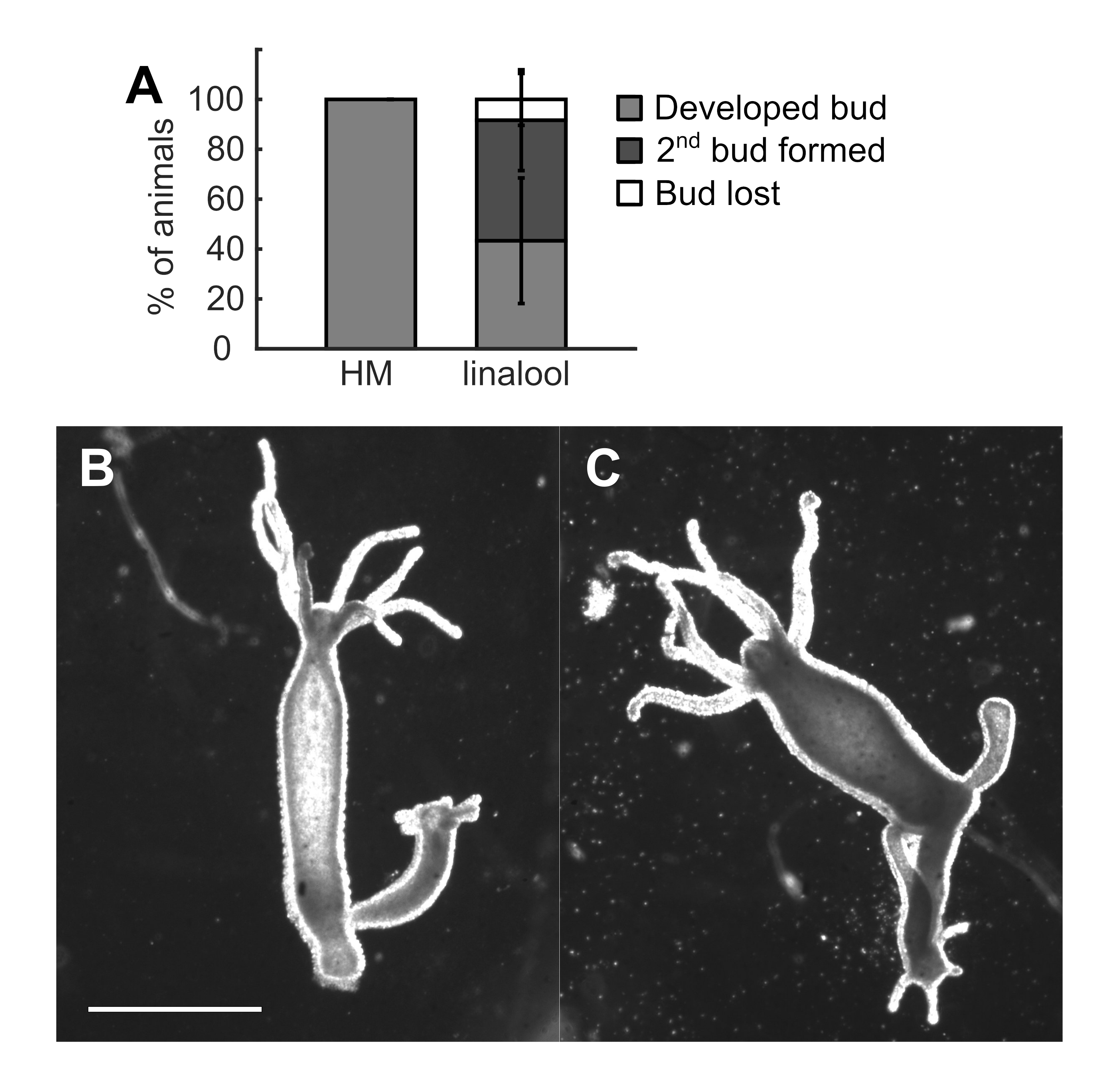

Supplement: S5 Fig — A. Bud development in budding animals incubated continuously for 3d in HM or 1 mM linalool, 30 animals per condition across 3 technical replicates. There was no statistically significant difference between animals in HM and in linalool (2-tailed t-test). Error bars represent SD. B. Representative image of animal with fully developed bud. C. Representative image of animal with two buds. Scale bar: 1 mm. (TIF) [file pone.0224221.s005.tif]

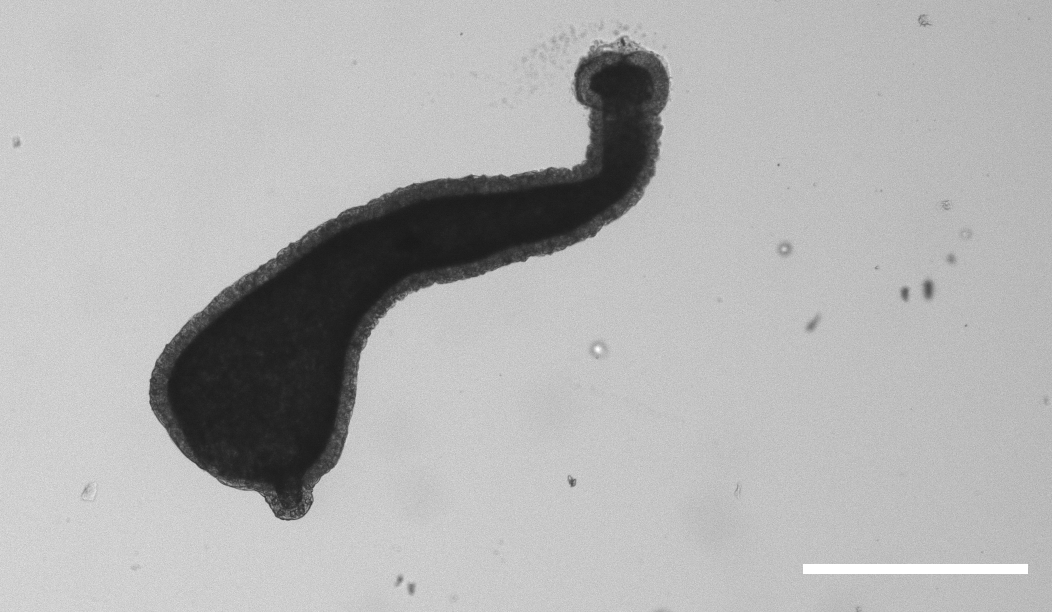

Supplement: S6 Fig — 14/42 decapitated polyps across 4 technical replicates regenerated small tentacle buds at the end of 3d incubation in linalool. Linalool solution was changed every day. The remaining animals did not regenerate head structures. Scale bar: 0.5 mm. (TIF) [file pone.0224221.s006.tif]

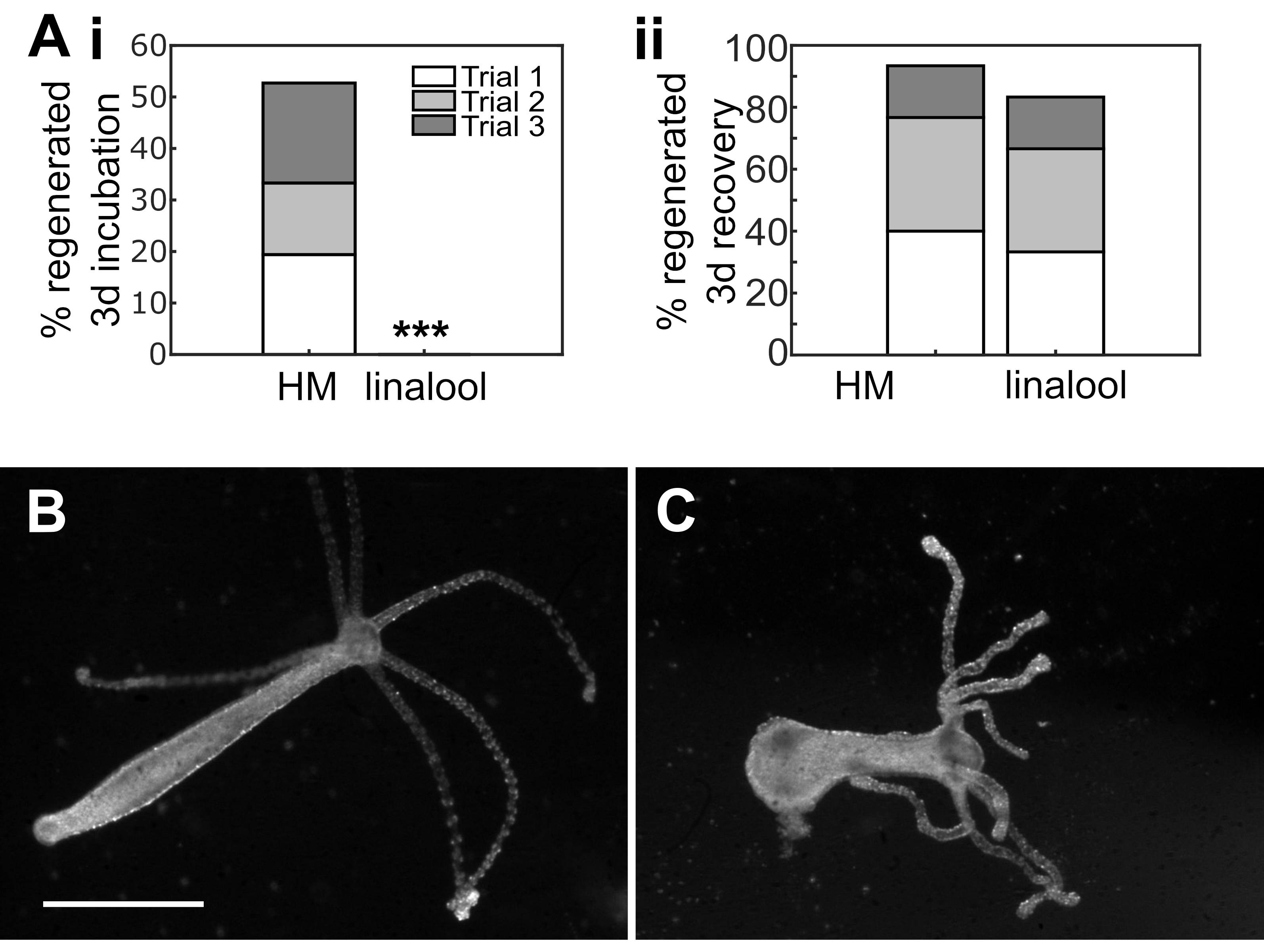

Supplement: S7 Fig — A. i. Three-day incubation in linalool prevents foot regeneration. Data from 36 polyps per condition across 3 technical replicates. (***) denotes statistically significant difference between percentage of animals with regenerated foot in HM and linalool at p < 0.001 (Fisher’s exact test) when comparing overall numbers. ii. Phenotype is rescued after 3d recovery in HM. Data from 30 polyps per condition across 3 technical replicates. B. Polyp incubated 3d in HM after foot amputation. C. Polyp incubated 3d in 1 mM linalool after foot amputation. Scale bar: 1 mm. (TIF) [file pone.0224221.s007.tif]

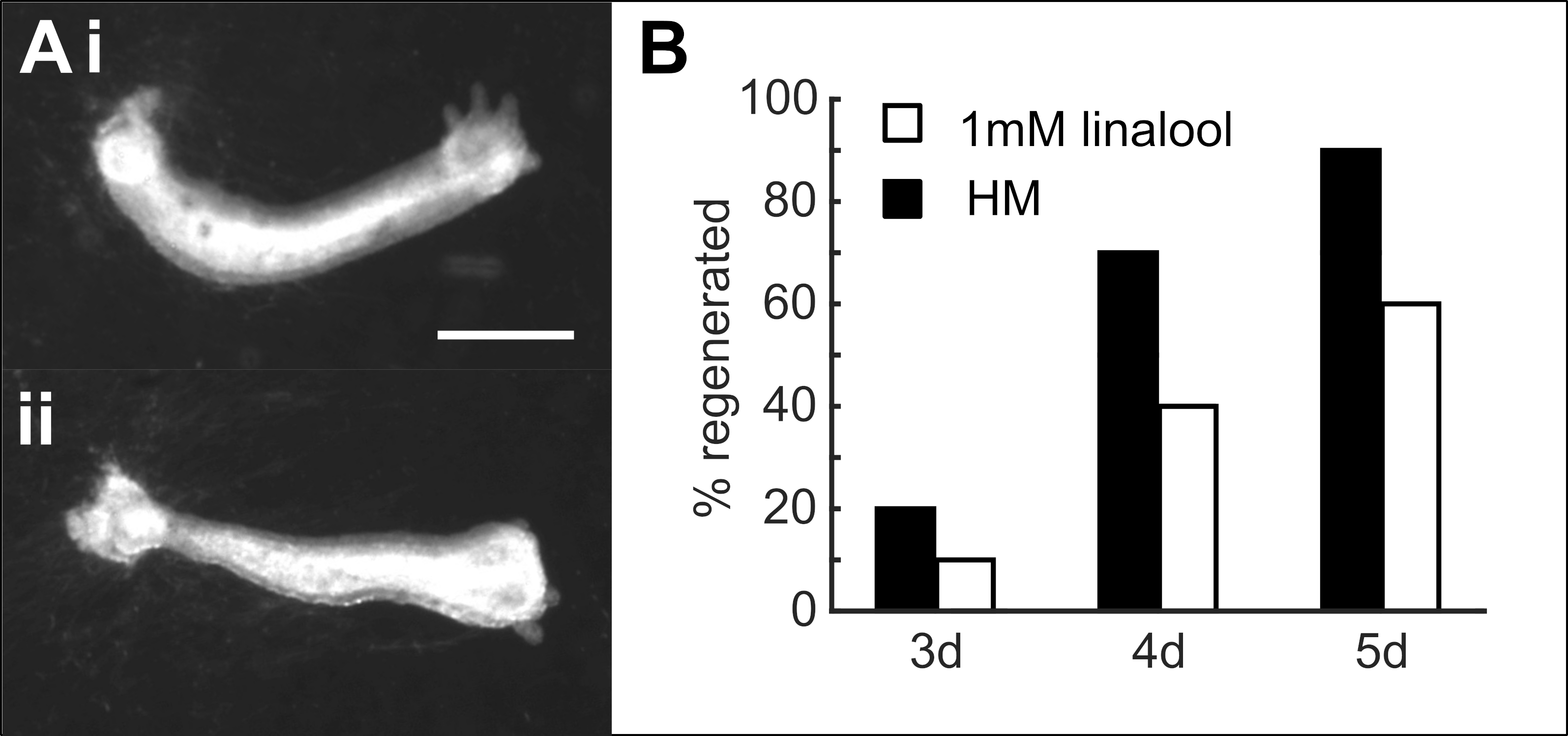

Supplement: S8 Fig — A. Representative images of nerve-free polyps regenerating their heads in i. HM and ii. linalool after 4d incubation. Scale bar: 1 mm. B. Percentage of animals with at least one regenerated tentacle over time (n = 10 animals in 2 technical replicates). There is no statistically significant difference between animals regenerating in HM compared to those regenerating in linalool (Fisher’s Exact test). (TIF) [file pone.0224221.s008.tif]

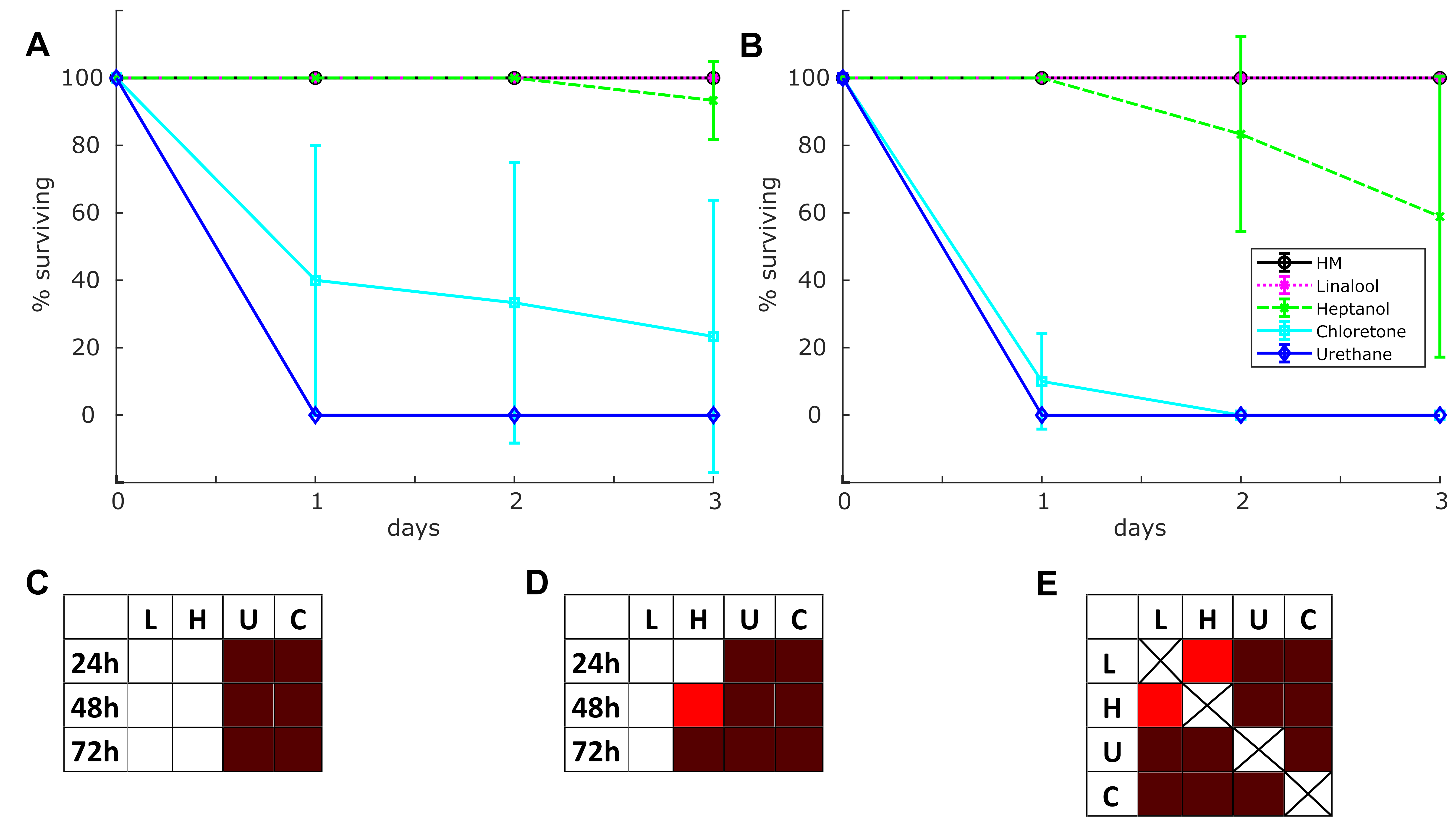

Supplement: S9 Fig — A. Incubation without changing media. n = 20 animals per condition across 3 technical replicates. Surviving heptanol and chloretone animals had a normal pinch response at 3d. Surviving linalool animals remained anesthetized. B. Incubation with media exchanged every 24h. n = 22 animals per condition across 3 technical replicates except for linalool and urethane where 2 technical replicates with 5 animals per replicate were performed. C. Statistical comparison of number of surviving animals at each time point in each anesthetic (without media changes) with the HM control as reference (Fisher’s Exact test). D. Statistical comparison of number of surviving animals at each time point in each anesthetic (with media changes) with the HM control as reference (Fisher’s Exact test). E. Pairwise statistical comparisons of number of animals surviving at the end of the 3d incubation in the anesthetics (with media changes) (Fisher’s Exact test). (C-E) Pink, red and dark red indicate a statistically significant difference at p<0.05, p<0.01 and p<0.001 respectively, determined using the Fisher’s Exact test between pairs of anesthetics. (TIF) [file pone.0224221.s009.tif]

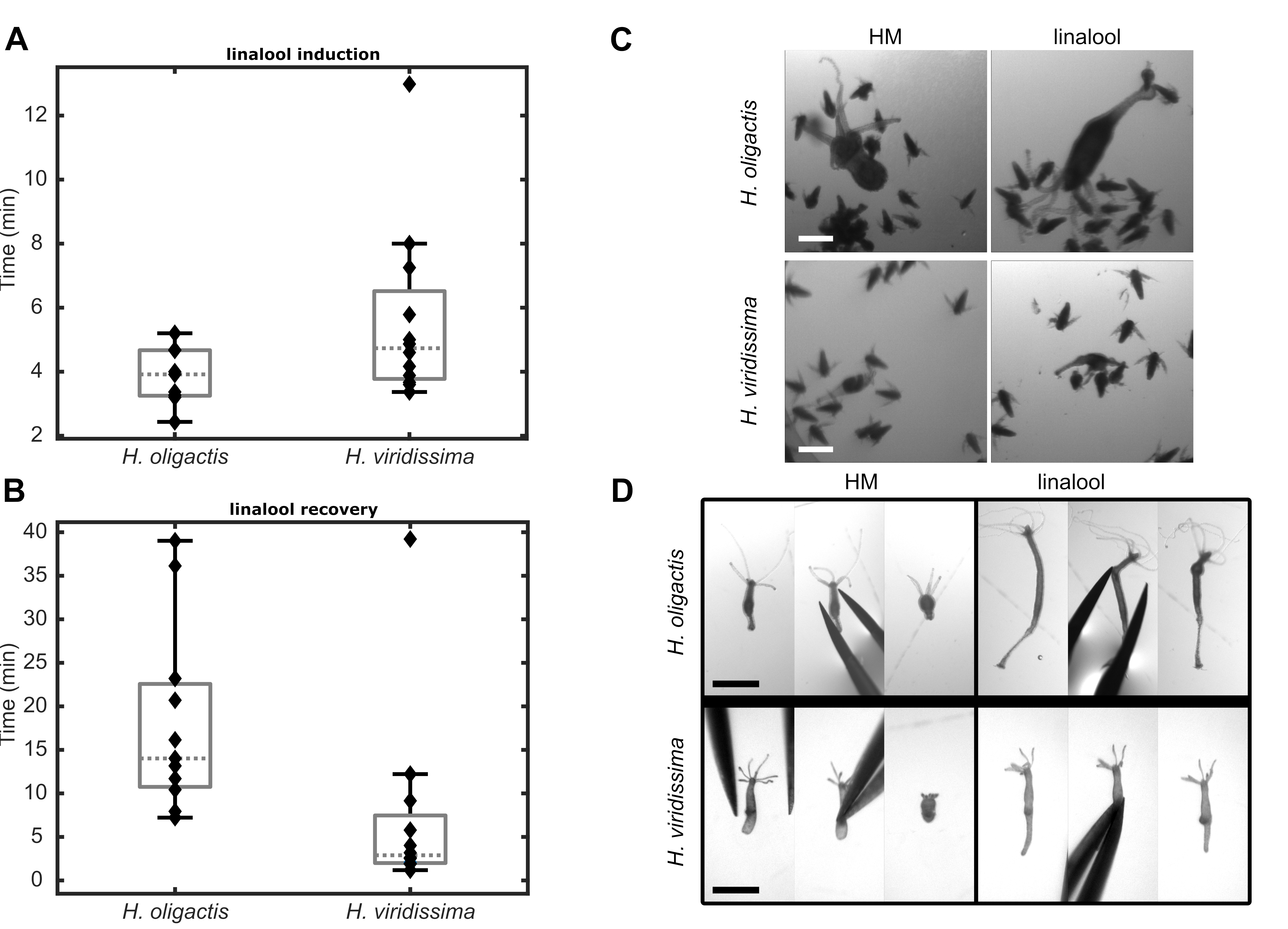

Supplement: S10 Fig — A. Time for induction of anesthesia, measured as time of full extension after last observed contraction burst, in 1 mM linalool (n = 9 for H. oligactis across 3 technical replicates, n = 12 for H. viridissima across 3 technical replicates). B. Time for recovery from anesthesia, measured as time of first observed contraction burst, after being moved to HM from 1 mM linalool (n = 11 for H. oligactis across 3 technical replicates, n = 12 for H. viridissima across 3 technical replicates). C. Feeding assay. Feeding is inhibited due to linalool incubation. While animals in linalool have shrimp stuck to their tentacles, they do not have any in their body column, contrary to what is seen with animals in HM. Scale bar 0.5 mm. D. Pinch responses in HM and 1 mM linalool. Pinch response is inhibited by linalool incubation. Scale bar 1mm. (TIF) [file pone.0224221.s010.tif]
