# Supplementary material for: Linalool acts as a fast and reversible anesthetic in Hydra
Source: PLoS One. 2019 Oct 24;14(10):e0224221. doi: 10.1371/journal.pone.0224221 (PMC6812832; doi:10.1371/journal.pone.0224221)
Supplement: S1 Table — Calculated for t-stacks of GCaMP animals imaged at 4x and z-stacks of GCaMP animals imaged at 60x, in 1 mM linalool and in HM. (**) indicates statistically significant difference from corresponding imaging in Hydra medium at p < 0.01 as determined by a two-tailed t-test. (DOCX) [file pone.0224221.s019.docx]

**Table S1: Coefficient of variation calculated for t-stacks of GCaMP animals imaged at 4x and z-stacks of GCaMP animals imaged at 60x.**, (**) **indicates statistically significant difference from corresponding imaging in Hydra medium at p < 0.01 as determined by a two-tailed t-test.**

|  | **Hydra medium** | | **1mM Linalool** | |
| --- | --- | --- | --- | --- |
|  | **4x** | **60x** | **4x** | **60x** |
|  | 0.222 | 0.152 | 0.154 | 0.136 |
|  | 0.168 | 0.199 | 0.145 | 0.149 |
|  | 0.195 | 0.158 | 0.143 | 0.099 |
|  | 0.176 | 0.183 | 0.147 | 0.113 |
|  | 0.163 | 0.268 | 0.139 | 0.144 |
|  | 0.172 | 0.170 | 0.137 | 0.084 |
|  | 0.163 |  | 0.143 |  |
|  | 0.148 |  | 0.142 |  |
|  | 0.202 |  | 0.127 |  |
|  | 0.137 |  | 0.156 |  |
| **mean** | **0.175** | **0.188** | **0.143**** | **0.121**** |
| **stdev** | **0.025** | **0.042** | **0.008** | **0.026** |
